# Supplementary material for: CRISPR-powered quantitative keyword search engine in DNA data storage
Source: Nat Commun. 2024 Mar 15;15:2376. doi: 10.1038/s41467-024-46767-x (PMC10943086; doi:10.1038/s41467-024-46767-x)
Supplement: Supplementary file 1 — Supplementary Information [file 41467_2024_46767_MOESM1_ESM.pdf]

Supplementary Information

CRISPR-powered Quantitative Keyword Search Engine in DNA Data Storage

Jiongyu Zhang[1,2], Chengyu Hou[1,2], and Changchun Liu[1]*

1. *Department of Biomedical Engineering, University of Connecticut Health Center, Farmington, CT 06030, United States*
2. *Department of Biomedical Engineering, University of Connecticut, Storrs, CT 06269, United States*

[*] **Corresponding author**

Dr. Changchun Liu

Department of Biomedical Engineering

University of Connecticut Health Center

263 Farmington Avenue

Farmington, CT 06030

Phone: (860)-679-2565

E-mail: chaliu@uchc.edu

1. Discussion of search scheme used in SEEKER

1.1 Search with long queries

By setting up multiple reactions to build a search array, it is possible to adapt SEEKER to search for longer keywords, phrases, and sentences. It is very likely that this kind of search array is built on grid-patterned microchambers, with each chamber pre-storing CRISPR reaction with an individual query as a split of the original long query. When in operation, the file amplicons may flush through the corresponding chamber row to enable mixing of the CRISPR reaction, crRNA query, and DNA target. A schematic draft of this system is shown in Supplementary Fig. 1a.

In some special cases, different phrases may contain word permutations where same words appear but in different orders. SEEKER is still able to identify word permutations in a phrase by searching the junctions of words, For instance, to distinguish “never give up” and “give up never”, we can use the query “er_giv” (or “ver_gi”) to confirm the presence of junction between words “never” and “give”, and use the query “up_nev” (or “p_neve”) to confirm the presence of junction between words “up” and “never” (“_” refers to blank space in phrases) (Supplementary Fig. 1b). The coding scheme used in SEEKER supports this way of searching as we encoded every two text symbols into a 7-nt sequence segment, instead of encoding each word into a fixed-length sequence. In the future, more analysis will be explored to evaluate its performance in a scaled-up system.

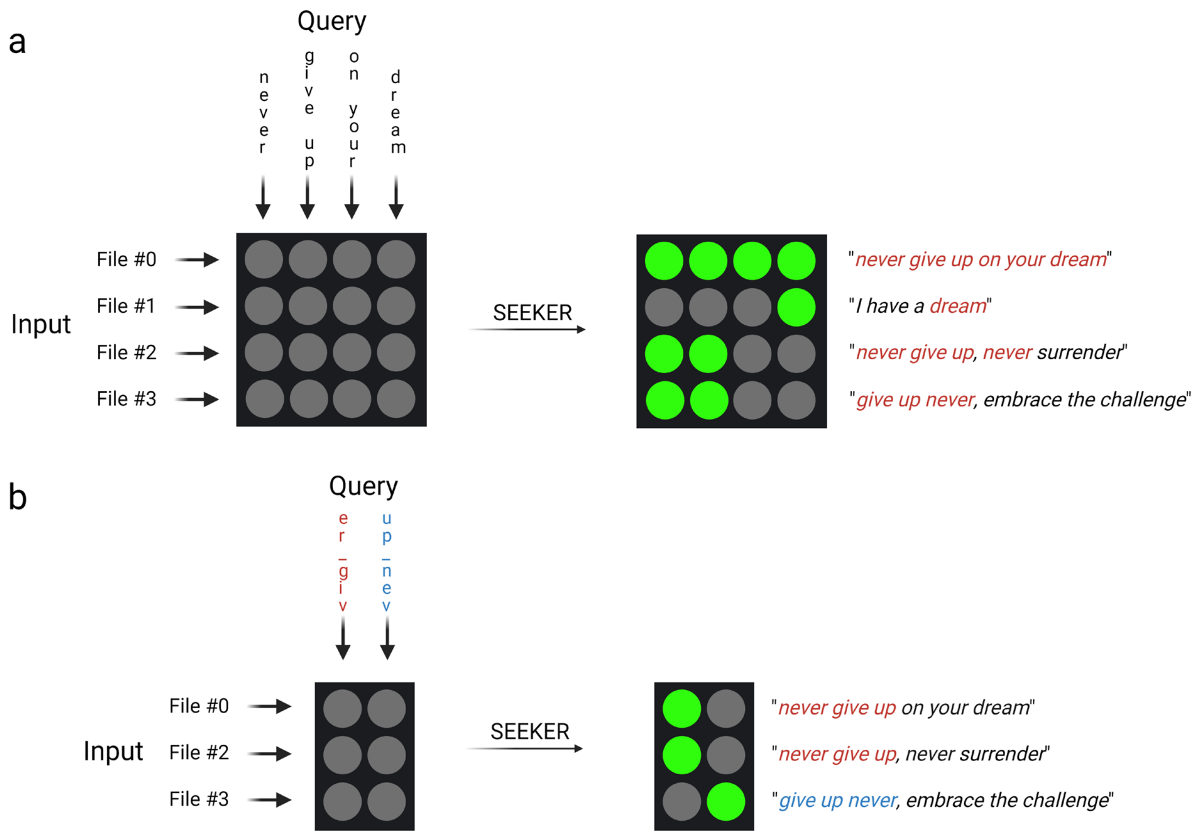

Supplementary Fig. 1. Searching with long queries in SEEKER. a, By setting up multiple reactions to build a search array, it is possible to adapt SEEKER to search longer keywords, phrases, and sentences. This search array can be created in grid-patterned microchambers pre-loaded with CRISPR reactions, with each individual short query as a split of the original long query. During operation, the file amplicons may flush through the corresponding chamber row, enabling mixing of the CRISPR reaction, crRNA query, and DNA target. The digital fluorescence state of 0/1 will indicate the presence or absence of short queries, allowing the user to infer the existence of a long query. **b,** The distinguishment of long queries with permutations of words can be achieved by detecting the "junctions" between words where word permutations differ from each other. For instance, by searching with the query "er_giv" (or "ver_gi") we can identify the existence of "never give up", while by searching with the query "up_nev" (or p_neve") we can identify "give up never".

1.2 Search efficiency

The search scheme we proposed in this work can be categorized as a simple linear search, which starts at a defined file and sequentially opens each file in the search scope to determine whether the keyword exists. In addition to this search method, there are other more advanced search algorithms employed in digital search engines that could potentially be used in molecular search systems. Here, we listed major existing algorithms and made a comparison regarding the complexity in Big-O notation.

Name of Algorithm	Complexity	Notes
**Inverted Indexing**	O(1) for exact term lookup<br>O(log(N)) for phrase queries	N is the number of documents containing the keyword
**TF-IDF (Term Frequency-Inverse Document Frequency)**	O(N × M)	N is the number of documents and M is the number of unique keywords in a query
**PageRank**	$O(V^3)$	V is the number of pages in the web graph
**Cosine Similarity**	O(N × M)	N is the number of documents and M is the number of unique keywords in a query
**B-Trees**	O(log(N))	N is the number of keys in the tree
**BM25 (Best Matching 25)**	O(N × M)	N is the number of documents and M is the number of unique keywords in a query
**SVD (Singular Value Decomposition)**	O(N × M × 2)	N is the number of documents and M is the number of unique keywords in a query
**Linear search (as demonstrated in this work)**	O(N × M)	N is the number of documents and M is the number of unique keywords in a query

Supplementary Table 1. List of major existing search algorithms employed in search engines and their complexity.

Although the method demonstrated in this work is more approximate to a simple linear search, it has quantitative search capability by counting keyword frequency, similar to TF-IDF. These two techniques share the same complexity. Moreover, our method is comparable in complexity to some widely used search algorithms that have the ability to return the relevance of documents to a query, such as Cosine Similarity, BM25 and SVD. In addition, the SEEKER system can adapt to the more efficient inverted indexing technique with a “metadata + file ID”

configuration, at little expense of additional memory storing the pointers, the metadata, and their relevance to the file. Herein, we proposed a supplemental SEEKER-based search scheme with oligos designed for inverted indexes, which is shown in Supplementary Fig. 2.

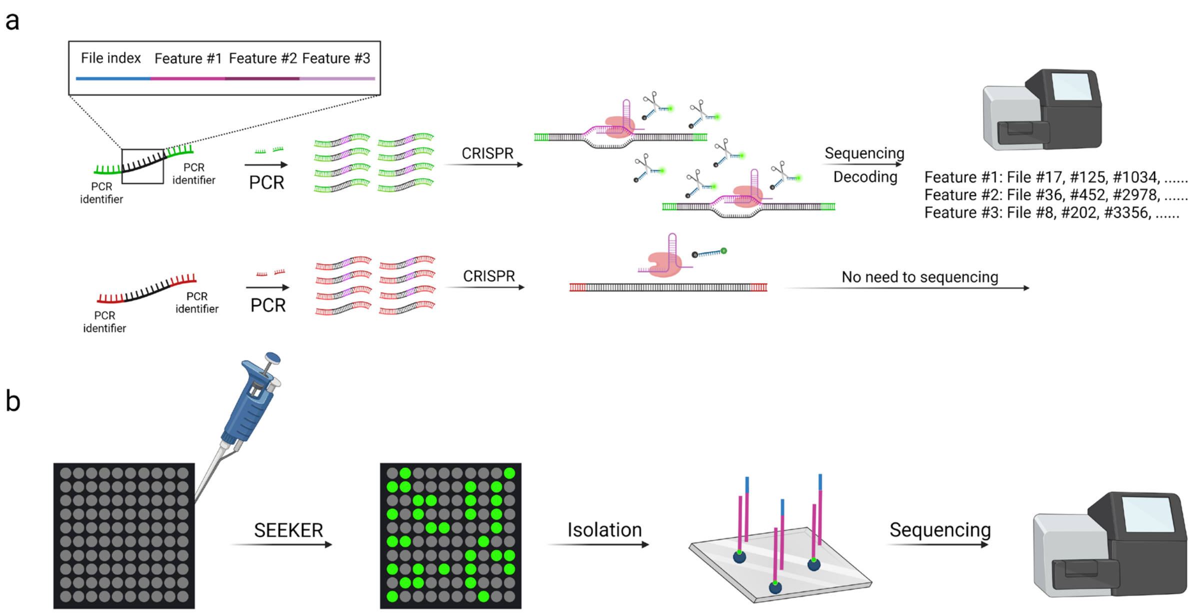

Supplementary Fig. 2. Envisioning SEEKER in more advanced search settings. a, In a metadata search scheme, the features of a file, as the metadata, are pre-computed and encoded in a DNA strand along with the file index to assemble the payload. Each oligo is prefixed and suffixed with a set of PCR primer targeting sites to enable enrichment of the file. SEEKER can still be applied in this condition. When the query crRNA recognizes a matching feature, there will be fluorescence response in this enriched oligo pool, and no fluorescence will be observed if no matching feature is found. For the enriched pool with fluorescence signals, we can sequence the original pool using a small volume, or we can directly sequence the CRISPR reaction after SEEKER. **b,** The combination of SEEKER and hybridization-based search approaches enables rapid determination of the physical location of files containing the keyword and isolation of the file, which could make molecular searching in DNA database more convenient and efficient.

2. Analysis and Calculation of Coding Potential

2.1 Background

To enable direct content searching of text data, the encoding of data should satisfy the following requirements:

1) The original order of words cannot be disrupted, otherwise the keyword search would not be achievable. This excludes block-sorting compression.
2) The search is meant to be implemented prior to reading the file. Therefore, any coding techniques in which the dictionary can only be obtained after reading the complete content of the file should be excluded. This excludes some widely applied dictionary coder such as Lempel-Ziv compression and its variants.
3) It is more beneficial to use fixed-length coding if we intend to conduct keyword search molecularly. On one hand, SEEKER naturally limits the length of a query sequence since the spacer of crRNA may only contain 18–24 nt. On the other hand, the proper length of a query is even more important if we adopt conventional strategies such as enzyme-free DNA hybridization. This is mainly because the length of a DNA strand will greatly affect its binding affinity and specificity. If a query contains very few bases, it may not bind efficiently to its target and thus may result in a search failure. If the query sequence is too long, the efficiency and specificity of binding may become uncontrollable due to a higher risk of strong secondary structure formation in the query, the potential for homodimers to form between queries, and non-specific interactions between the query and irrelevant nucleic acids. This excludes Huffman coding, arithmetic coding, and their variants.
4) The query sequences should not be different across a single data block if it refers to the same keyword. This excludes run-length encoding, which has been widely applied to DNA data storage.

The general idea of this work is to provide an algorithm allowing for direct mapping of a query sequence through a small portion of the entire oligo pool, which functions as the dictionary. This is done while keeping each symbol or symbol combination stored in a fixed-length code, ensuring the arrangement of raw data is not distorted. More importantly, the overall cost of writing data and reading data-of-interest after searching should be kept as low as possible. The algorithm we present here is named "non-collision grouping" (NCG) coding. The method is based on finding fragments

of data with no "collisions" (a "collision" occurs when binary "1"s appear at the same bit position among different data fragments) and grouping them together. In this way, the data can be compressed by assigning repeated fragments into the same group, and the dictionary shrinks since one group may contain more than one data fragment.

2.2 Definitions

Some definitions of the basic concepts mentioned in the following analysis need to be clarified before moving forward. This algorithm intends to map data written in plain text form to DNA sequences, which are divided into two categories: reference strands and data strands. The list of positions of binary "1"s in a data fragment is called a "combination". A set of "combinations" without "collisions" are defined as a "group", whose information is stored in reference stands. The data strands store data in its original arrangement. In a data strand, every short oligonucleotide sequence converted from a data fragment is called a data unit, which consists of several "index bases" and one "pointer base". All reference strands make up a reference pool and all data strands make up a data pool.

2.3 Analysis

Assume the total number of symbols being encoded is N. Since the symbols are in plain text (UTF-8) format, every symbol is represented by an 8-bit binary number and the size of the data can be written as: $S = 8N$. The grouping interval refers to the length of the binary data encoded in one unit. We define the grouping interval as $G = 8n_1$, where n_1 is an arbitrary integer representing the number of characters being encoded in one unit. In a data strand, we define the length of an oligo fragment encoding one unit or a single element in the groups as n_2. Considering there is one base for "pointer", the actual space for indexing is $L_{dpi} = n_2 - 1$.

The total number of units in the data pool is:

$$U_{dp} = \frac{S}{G} = \frac{\mathrm{N}}{\mathrm{n}_1} \tag{1}$$

The total number of bases making up the payload of the data strands is:

$$B_{dp} = U_{dp}n_2 = \frac{N}{n_1}n_2 \tag{2}$$

The number of reference bases is determined by the quantity of “non-collision” groups, which can be represented as:

$$B_{rp} = GN_{dpi} = 8n_1 \cdot \text{len(groups)} \tag{3}$$

Under a certain data size, the number of “non-collision” groups will directly influence the coding potential by influencing the number of reference bases and the length of a data unit. Therefore, we determined the upper and lower limits of coding potential by estimating the upper and lower bounds of the number of groups, both from a theoretical perspective based on the consideration of extreme conditions of data composition, and from a practical perspective based on a probabilistic model with real input text data.

Considering the extreme conditions, the best-case scenario is when the data only contains three unique units, and these units happen to be “non-collided” and are therefore assigned to one group. The worst-case scenario is when the data contains no repeated units and no “non-collision” units are found, and thus each group only includes one data unit.

In the worst-case scenario, suppose we have N symbols, making up $8N$ bits of data in total. Given the grouping interval $G = 8n_1$, if all the data units are unique, the number of groups should be:

$$N_G = \frac{8N}{8n_1} = \frac{N}{n_1} \tag{4}$$

Then, the number of bases in the reference strands should be the same as the data size:

$$B_{rp} = 8n_1 \cdot \frac{N}{n_1} = 8N \tag{5}$$

The number of bases in the data strands will be:

$$B_{dp} = \frac{8N}{8n_1} \cdot (\log_3 N_G + 1) = \frac{N}{n_1}\left(\log_3 \frac{N}{n_1} + 1\right) \tag{6}$$

As this analysis is for theoretical estimation of the upper and lower limit of the coding potential, we assume the number of data bases to encode one data unit changed continuously rather than by one base if the threshold is reached, and the encoding volume n_2 is no longer considered in the following analysis.

Considering each binary code has its non-collided "complement code" (i.e., "00000100" and "11111011"), the ASCII code table containing 256 symbols can be divided into two parts, the original codes and the complement codes, in which each original code can pair with its complement code and be assigned to one group, but the original codes can only be assigned to different groups. In this case, when the grouping interval is 8 ($n_1 = 1$), which means one data unit contains one ASCII code, there are $2^7 = 128$ original codes and 2^7 groups in maximum. When the data size is below or equal to 2^7 bytes, in the worst-case scenario we assume the data is all composed of original codes. Then, the coding potential when ($N \leq n_1 \cdot 2^7 = 2^7$) can be calculated as:

$$P = \frac{8N}{8N + N(\log_3 N + 1)} \tag{7}$$

When the data size is larger than 2^7 bytes, which refers to $N > 2^7$, the coding potential is:

$$P = \frac{8N}{8 \times 2^7 + N(\log_3 2^7 + 1)} \tag{8}$$

Similarly, when the grouping interval is 16 ($n_1 = 2$), there are 2^{15} original codes. When $N \leq n_1 \cdot 2^{15} = 2^{16}$, the representation of the coding potential is:

$$P = \frac{8N}{8N + \frac{N}{2}\left(\log_3 \frac{N}{2} + 1\right)} \tag{9}$$

When $N > 2^{16}$, the coding potential is:

$$P = \frac{8N}{8 \times 2^{16} + \frac{N}{2}(\log_3 2^{15} + 1)} \tag{10}$$

We can generalize the representation of the coding potential with any n_1 as:

$$P = \begin{cases} \dfrac{8N}{8N + \dfrac{N}{n_1}\left(\log_3 \dfrac{N}{n_1} + 1\right)}, & 0 < N \le n_1 \cdot 2^{8n_1 - 1} \\ \dfrac{8N}{8n_1 \cdot 2^{8n_1-1} + \dfrac{N}{n_1}(\log_3 2^{8n_1-1} + 1)}, & N > n_1 \cdot 2^{8n_1-1} \end{cases} \tag{11}$$

In the best-case scenario, we assume the data are ideally composed of repeats of three "non-collided" data units, which can be exactly assigned to one group, resulting in only $8n_1$ bases in the reference strand. Then, the coding potential can be represented as:

$$P = \frac{8N}{8n_1 + \dfrac{2N}{n_1}} \tag{12}$$

Based on the above analysis, we can then determine the lower and upper bounds of the coding potential as a function of symbol number N and grouping interval n_1. With data size as the X-axis, the upper and lower bounds of the coding potential under different n_1 are plotted in Supplementary Fig. 3a.

As the best-case scenario reflects an ideal condition that may rarely happen with real-world data, we speculated that the worst-case scenario may be more approximate to actual coding potentials. Through comparison of the lower theoretical bounds, we were able to determine the best grouping interval and the range of data block sizes to be applied in NCG coding. With the current stage of DNA data storage mostly involving data sizes at the MB level, we calculated the lower bound of coding potential under different n_1 with data sizes up to 1,000 MB (Supplementary Fig. 3b). We found in the cases of $n_1 = 1$ and $n_1 = 2$, the lower bound rapidly grew when the data size was less than 1 MB. When $n_1 = 2$, the lower bound was the highest across the studied data size range. Finally, when $n_1 = 3$ the lower bound gradually increased but was still slightly lower than the case of $n_1 = 2$ when the data size reached 1,000 MB. For higher n_1 values, the lower bounds remained significantly lower. This result suggested that $n_1 = 2$ might be a better choice as the grouping interval for NCG for text data encoding.

Then, we aimed to provide measured upper and lower bounds based on a probabilistic model using real input data, which can better reflect the actual conditions of text data encoding. To demonstrate this, we downloaded ~100 MB of text data from Wikipedia as the input. The

original data were divided into multiple blocks with a certain size, and different block sizes were studied to reveal the relationship between data size and the number of groups under the NCG algorithm. For each block size, 100 blocks were computed as experimental repeats, and we expect the number of groups in these 100 experiments to follow a normal distribution. This allows us to estimate the upper and lower limits from a probabilistic perspective. The block sizes chosen were 5 KB, 10 KB, 25 KB, 50 KB, 100 KB, 250 KB, 500 KB, and 1,000 KB.

Before moving on to the measurement of the number of groups, we aimed to confirm the conclusion drawn from the theoretical analysis that $n_1 = 2$ should be applied to NCG coding. We analyzed the coding potential when the n_1 value ranged from 1 to 3 using real text data from Wikipedia, testing 10 different blocks as replicates for each size. We confirmed that $n_1 = 2$ had a better actual coding potential with data sizes below 50 KB (Supplementary Fig. 3c). When the data size further increased, although the coding potential for $n_1 = 3$ might increase, the number of groups would increase even more dramatically, resulting in much longer computation times and greatly reducing the efficiency. Therefore, we believe it is reasonable to use $n_1 = 2$ as the grouping interval.

With the grouping interval n_1 determined as 2, the distributions of the number of groups are shown in Supplementary Fig. 3d, with block sizes marked in each subfigure. For most block sizes, the number of groups in the 100 experiments followed a normal distribution. The measured results of the number of groups are also shown in Supplementary Fig. 3e. We observed an increase in the average number of groups as the block size increased, showing a seemingly linear relationship with the logarithm of block size. However, there was substantial variation in the number of groups when the block size became large, which led to an even fewer number of groups for a larger block size in some cases.

We define the upper and lower limit as when the significance level reached 0.01, where the probability of observing a number of groups exceeding the defined upper limit or below the defined lower limit is 0.01. To calculate the upper and lower limit, we used the Z-score method:

$$Z = \frac{x - \mu}{\sigma} \tag{13}$$

where x is the observed value, μ is the sample mean, and σ is the standard deviation. From the Z-score table, the Z-value should be 2.33 for a significance level of 0.01. Thus, the upper and lower limit are calculated as:

$$x_L = \mu \pm 2.33\sigma \tag{14}$$

The upper and lower limit of the number of groups with every block size tested are shown in Supplementary Fig. 3f. The upper and lower limits rapidly increased when the data size was smaller than 100 KB. When the data size further increased, the lower limit reached a stable value at around 1,000, while the upper limit still increased but at a slower rate. We anticipate that the upper limit would soon reach its threshold if the data size were further expanded, due to the fact that the number of frequent character combinations in the English text is limited. We also calculated the practical upper and lower limit of coding potential for every block size we tested based on the estimated upper and lower limit of the number of groups. As shown in Supplementary Fig. 3g. the overall performance was significantly better than that of the conventionally used homopolymer-free base mapping[1] which had a theoretical coding potential of $\log_2 3 \approx 1.58$ bits/nt. In some cases, the upper bound of coding potential exceeded 2 bits/nt, which was even higher than that of the simple mapping of two binary digits to one nucleobase (e.g., "00" to "A", "01" to "G", "10" to" C" and "11" to "T") not considering avoidance of homopolymers, indicating effective compression of the original data. It should be noted that in practice, when the number of groups exceeds $3^6 = 729$, an additional base must be added to each data unit of the data strands, which may compromise the overall coding potential. Consistent with our speculation, the optimal upper limits of the coding potential were obtained with data sizes of 25–50 KB, which corresponded to a measured number of groups close to 729 (Supplementary Fig. 3e). As the data sizes further enlarged, the coding potential dropped back to below 2 bits/nt but kept approaching 2 bits/nt. We concluded that in this coding scheme, to achieve a higher coding potential the data size encoded with one dictionary should be limited to 25–50 KB, and more dictionaries are recommended if encoding data with larger sizes than the optimal range.

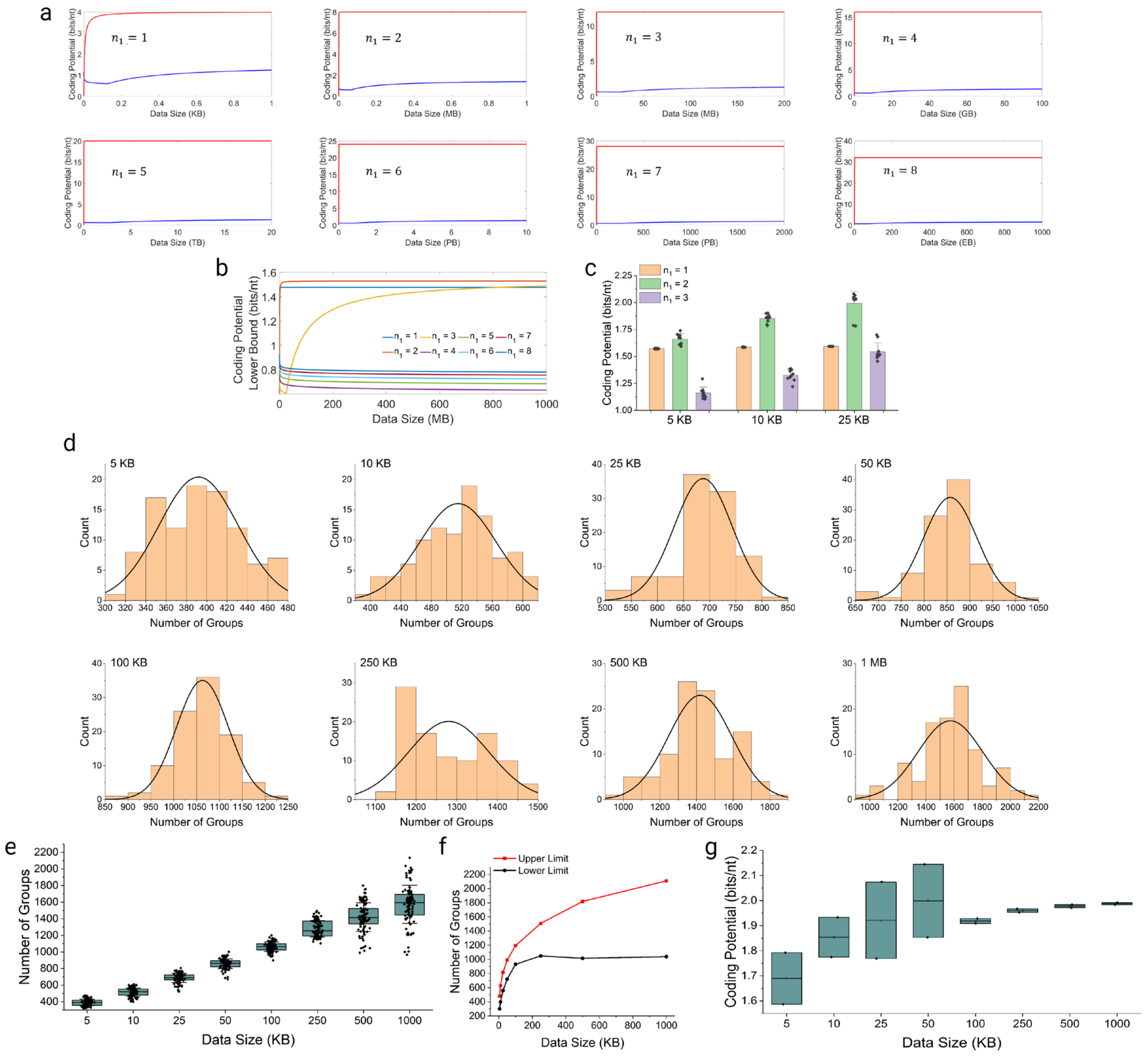

Supplementary Fig. 3. Theoretical and practical estimation of the coding potential for NCG. a, Theoretical upper (red curves) and lower (blue curves) bounds of the coding potential under different n_1 based on consideration of extreme conditions of data composition. The value of n_1 is marked in each subfigure. **b,** The lower bound of the coding potential when $n_1 \leq 8$ with data sizes up to 1,000 MB. **c,** The actual coding potential obtained with real text data from Wikipedia when $n_1 = 1, 2, 3$ ($n = 10$ different blocks. Data are presented as mean ± SD of 10 independent experiments). **d,** The normal distribution of the number of groups when $n_1 = 2$. Block sizes are marked in each subfigure. For each block size, 100 different blocks were tested as experimental replicates. **e,** The measured results of the number of groups ($n = 100$ different blocks. Data are presented as mean ± SD of 100 independent experiments). **f,** The calculated upper and lower limit of the number of groups for every block size based on a probabilistic estimation of normal

distribution. **g,** The upper and lower limit of coding potential for every tested block size based on the estimated upper and lower limit of the number of groups when $n_1 = 2$. The center line represents the median value and the bounds of the box indicate the upper and lower limit of coding potential. Source data are provided as a Source Data file.

2.4 Discussion

The NCG algorithm is a variant of the log|M'|/log(b) method, where |M'| refers to the number of unique binary digit combinations and b is the number of symbols in the coding alphabet. Since we used homopolymer-free base mapping as demonstrated by Goldman et al.[1], which converted binary codes to base-3 digits for mapping to nucleobases, the value of b is 3. If we adopt a similar data structure in the assembly of reference strands and data strands, the only difference between these two methods will be the use of "pointer" bases. For the NCG method, the "pointer" base is necessary, as different "pointer" bases refer to different data units in one group. Without the information of the "pointer" base, there is no way to retrieve the data. With the log|M'|/log(b) method, the "pointer" base can be eliminated from the data strands if we pre-define a "pointer" base or introduce a rule to map the "pointer" base, which applies to the retrieval of data units in all the groups.

When using the log|M'|/log(b) method, more bases will be in the reference strands since the number of groups (M') is greater or at least equal to what is generated by NCG algorithm. If we choose to store the dictionary along with the actual data in DNA, every time a portion of the oligo pool must be sequenced in order to determine the query sequence, allowing the searching to proceed. We have proved that to achieve the maximal overall coding density, one dictionary can only cover ~25–50 KB of data. This indicates that if we have a larger amount of data, multiple dictionaries are needed and all of them should be sequenced before molecular searches. Given the sequencing of the dictionary inevitable, we aim to minimize the cost of dictionary sequencing, and fewer reference bases undoubtedly become more desirable. This becomes an advantage of using NCG instead of the log|M'|/log(b) method.

To validate this assumption using experimental data, we used the data blocks from Wikipedia as used in Section 2.3 for practical coding potential analysis. We used both NCG and

the basic log|M'|/log(b) method to encode the data blocks and calculated the average number of reference bases per KB of data for different data sizes. As shown in Supplementary Fig. 4a, we found that there were always fewer reference bases for NCG, and the reduction of reference bases was more significant for larger datasets. We then calculated the reduction of reference bases in percentage and found that larger datasets led to a more significant reduction of reference bases for a data size up to 200 KB. The reduction reached its limit at ~15% for larger data sizes (Supplementary Fig. 4b). We also obtained the overall coding potentials for both methods and acknowledged that the basic log|M'|/log(b) method typically had a higher overall coding potential, except in some cases where the number of groups for the basic method exceeded the encoding volume ($V = 3^{n_2}$, where n_2 is the number of bases in one data unit) and had to include one more base in the data unit, while the number of groups for NCG was still within the range (Supplementary Fig. 4c).

However, we were more interested in assessing the effectiveness of these two approaches in terms of the cost of oligo synthesis and sequencing. We aimed to investigate whether, in cases where the sequencing of reference bases becomes inevitable, a reduced need for reference base sequencing will lead to an overall reduction in the cost of DNA data storage. With advancements in microchip technology, the cost of synthesis per nucleotide can potentially be reduced to $0.00001.[2] According to online data (https://ourworldindata.org/grapher/cost-per-gigabase-dna-sequencing), the cost of sequencing per gigabase was $5.04 in 2021. Based on these statistics, we can roughly estimate the overall cost of writing and reading data in DNA using both the NCG and log|M'|/log(b) methods. We investigated a case where 1 MB of data is stored in one dictionary, resulting in a reduction of reference bases to ~15% when using the NCG method. A previous study reported that information with a density of 215 PB/g can be recovered through sequencing[3]. Assuming a yield of 10 ng for the oligo pool and that the product from first PCR can be used at least once as the substrate in a new PCR without significant quality reduction, since each PCR requires only 0.00465 ng to access 1 MB of data, the original oligo pool can be used $2,150^2$ times without the need to synthesize a new pool. We have summarized the costs of synthesis and sequencing for these two methods in the following table.

Method	Coding Potential (bits/nt)	Synthesis Cost per MB	Average Reference Base per MB	Reference Sequencing Cost per MB ($2{,}150^2$ times)	Data base per unit	Data Sequencing Cost per MB ($2{,}150^2$ times)
**NCG**	2.0	$40	25,180	$586.74	8	$93189.6
**log|M'|/log(b)**	2.3	$34.78	29,318	$683.0	7	$81540.9

Supplementary Table 2. Statistics of the cost of synthesizing and sequencing data stored in DNA with the NCG method and the basic log|M'|/log(b) method.

Since the aim of searching is to find the content-of-interest, which is expected to take up only a portion of the entire dataset, we define the ratio of oligos encoding the content-of-interest as the x-variable. We assume only this portion of data bases is sequenced. Using the statistics in the table, we calculated the overall cost per MB of data as a function of x (Supplementary Fig. 4d). The point of intersection between the lines represents the point at which the costs for these two methods are equal. We found that when the ratio of oligos encoding the content-of-interest was below 0.78%, the NCG method had an overall lower cost compared to the log|M'|/log(b) method. In practical conditions, it is common for only a very small fraction of oligos in the entire oligo pool is expected to be sequenced. Taking Wikipedia as an example, there are only 11 words with a frequency ratio higher than the threshold of 0.78% (https://www.thingsmadethinkable.com/item/words_on_wikipedia.php). These words mostly consist of determiners such as "the", prepositions such as "of", "in", "to", "on", and "for", and conjunctions such as "and" and "as", which are not suitable for use as keywords. Therefore, assuming that the occurrence of words is evenly distributed throughout the Wikipedia corpus, the ratio of files containing a particular keyword will certainly be lower than 0.78%. This information suggests that NCG may be a better choice for achieving a lower cost in the complete writing-reading-searching process in DNA data storage.

Moreover, we aimed to demonstrate several more examples to extend the application of NCG from texts to pictures in JPG format and animation in GIF format, as well as some randomly generated binary codes. The encoded files are listed in Supplementary Data 8. To encode all the files, we applied an 8-bit grouping interval, considering every 5 KB of data as a segment or block for encoding. We calculated the number of reference bases per KB and the overall coding potential.

As shown in Supplementary Fig. 4e, the number of reference bases in the NCG algorithm reduced to about 60% of what was generated through the basic log|M'|/log(b) algorithm. This means that if it becomes inevitable to recover the dictionary for a file, the bases that need to be sequenced under the basic method will be 1.6 times of those under the NCG method, greatly increasing the sequencing price and time cost. As shown in Supplementary Fig. 4f, with certain block sizes the coding potential for NCG was even slightly higher than that of the basic method, although one more "pointer" base was consumed. This is because the reduction in the number of reference bases was more significant than the reduction in the number of data bases when a proper grouping interval was applied. This phenomenon is independent of the data format and serves as evidence that NCG benefited not solely from the sparsity of the English vocabulary and the repetition of common letters and words, but also from the "non-collision grouping" mechanism.

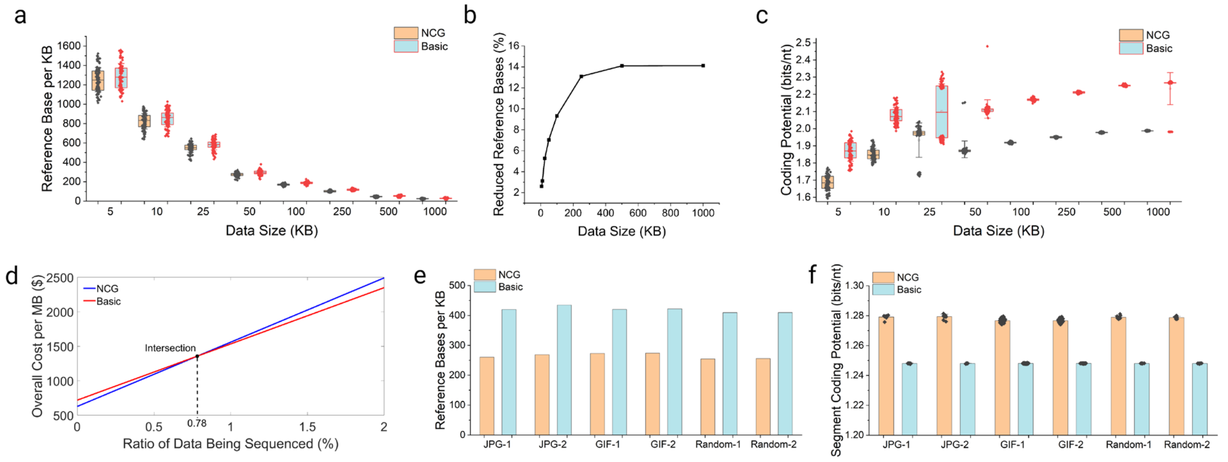

Supplementary Fig. 4. Comparison of NCG and the basic log|M'|/log(b) method. a, The average number of reference bases per KB of data with NCG and the basic log|M'|/log(b) method for different data sizes ($n = 100$ different blocks. Data are presented as mean ± SD of 100 independent experiments). **b,** The reduction of reference bases in percentage for NCG compared with the log|M'|/log(b) method. **c,** The calculated coding potential with both methods for different data sizes ($n = 100$ different blocks. Data are presented as mean ± SD of 100 independent experiments). **d,** The overall cost per MB of data as a function of the ratio of oligos being sequenced in the entire oligo pool. The intersection where the costs for NCG and the basic log|M'|/log(b) method were equal is marked. **e,** The number of reference bases per KB of data for both methods when the data was in non-text format. **f,** The coding potential for each 5 KB block of non-text data with both methods ($n = 9$ different blocks for JPG-1, $n = 11$ different blocks for JPG-2, $n = 135$ different blocks for GIF-1, $n = 61$ different blocks for GIF-2, $n = 10$ different blocks for Random-1 and

n = 10 different blocks for Randm-2. Data are presented as mean ± SD of independent experiments). Source data are provided as a Source Data file.

3. Algorithm Flowcharts

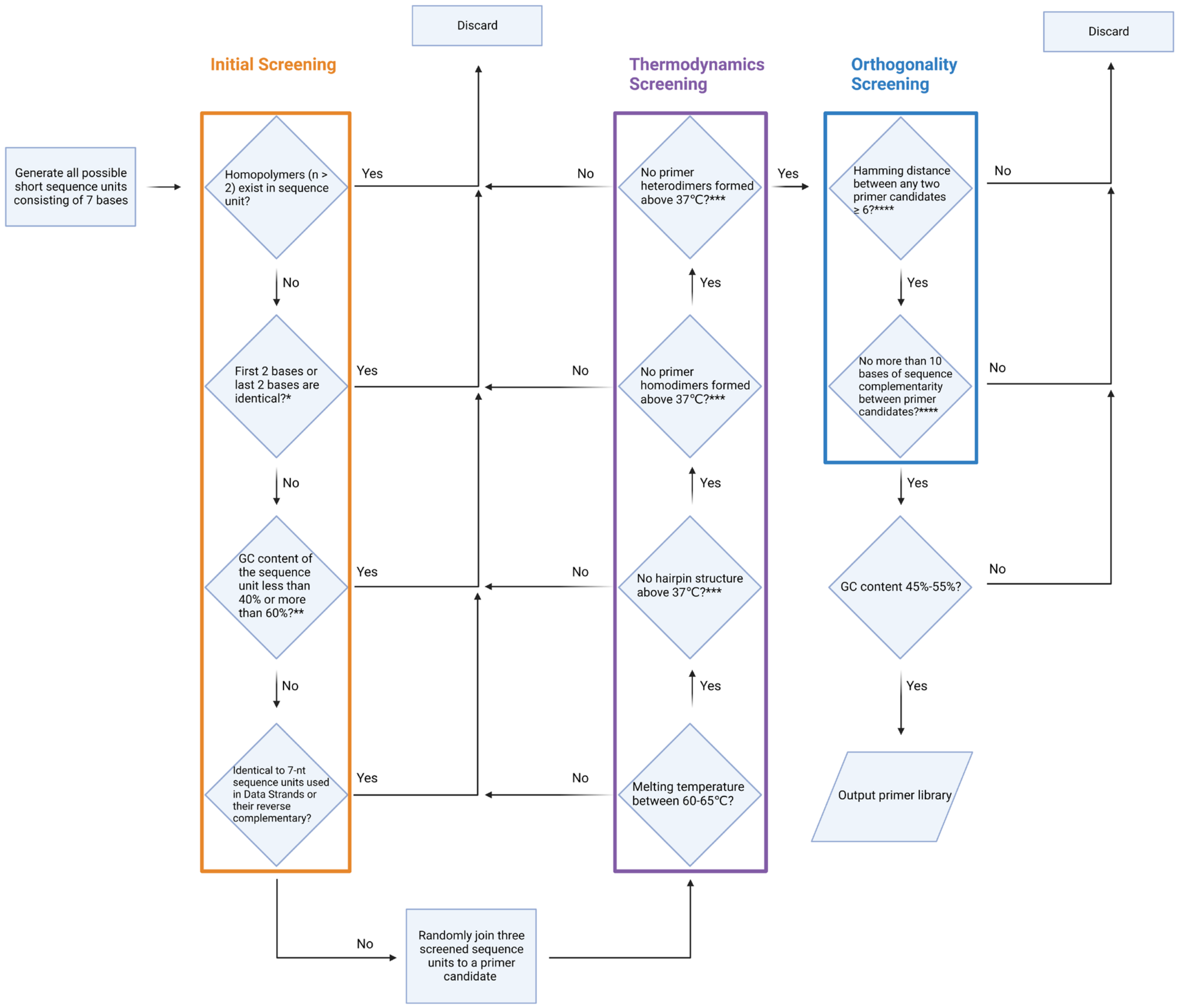

Supplementary Fig. 5. Step 0: PCR primer design. The PCR primers used in the reference pool and data pool were designed to contain 21 nucleotides, which was the same as the length of the query sequence in the crRNA spacer. The aim of this algorithm is to ensure that among the primers selected, no primer sequence is identical or very similar to a query sequence or its reverse complementarity, otherwise a mistake may occur when performing CRISPR-based searching. This criterion also avoids undesired amplification from the query sequence of a random data strand. Moreover, the design of primers should follow the basic rules in sequence design for DNA data storage, which includes: 1) being homopolymer-free and 2) having balanced GC content. Here, we generated short 7-nt sequence units that had GC contents between 40% and 60% with no homopolymers ($n > 2$) present when joining any three of these units to a 21-nt sequence. Then, the sequence units that are identical to possible sequence units in data strands were removed, and the remaining units were randomly joined to form a 21-nt sequence (each unit can only be used once to avoid

wide overlaps among potential primers). Assisted by Primer3[4,5], the generated primer candidates were subjected to a series of thermodynamics screening and sequence similarity screening as shown in the flowchart. The preserved ones made up the primer library, from which all PCR primers for the reference pool and data pool were selected. Any two primers from the primer library should have no crosstalk and ideally no interferences will occur if multiple primers are present in one reaction. In the thermodynamics screening steps, the parameters used for Primer3 predictions were 2.5 mM for the divalent cation concentration and 4 μM for the DNA concentration. These parameters were consistent with the experimental conditions. *If the first two bases or last two bases are identical, then a homopolymer with three or more identical bases would occur once joining these sequence units together. **To reduce the number of inputs for Primer3-based screening, every sequence unit was designed to have a balanced GC content. In this way, the final primer sequence should also be GC-balanced. As one sequence unit contains 7 bases, an ideal GC content of 45%–55% would result in 3.15–3.85 GC bases, which does not include any integers. Therefore, we expanded the range to 40%–60%. ***Though the annealing temperature for PCR was 55°C in this work, we applied more stringent screening criteria and set the temperature threshold as 37°C when checking the formation of hairpins, homodimers, and heterodimers among the primer candidates. ****The similarity screening criteria were obtained from Organick et al.[6] to prevent crosstalk between primers as much as possible.

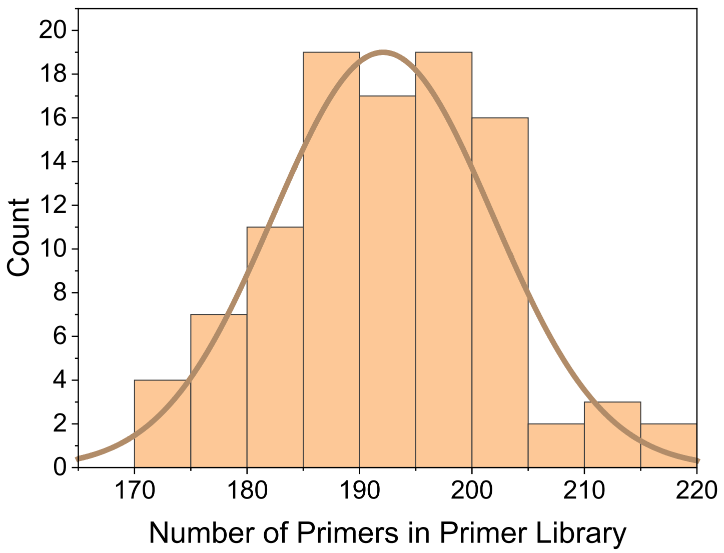

Supplementary Fig. 6. The distribution of quantities of primers in the primer library. After 100 trials of primer screening, the number of primers selected to the primer library ranged 170–219 and most likely centered around 185–205. The average number was 192.1. This implies that if one file consumes two primers, at most 109 files with unique IDs can be stored in one physical location of a DNA-based storage medium. Source data are provided as a Source Data file.

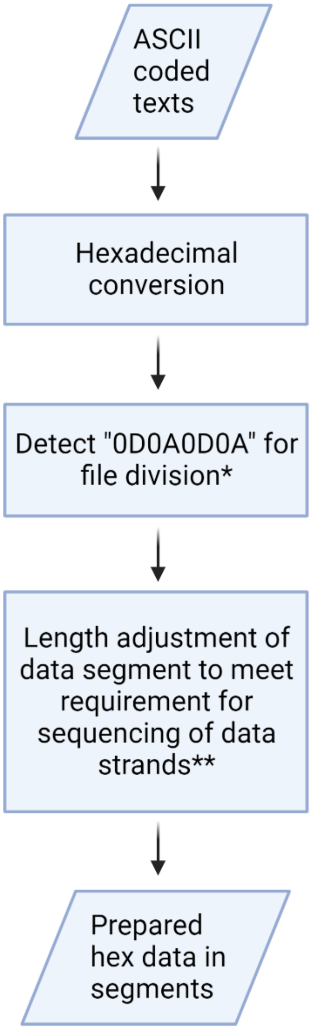

Supplementary Fig. 7. **Step 1: Pre-processing of text data.** *The original text file may contain many data segments that need to be losslessly stored. In this example, the input file contains 40 abstracts of journal articles, which are separate paragraphs in the text. Every two paragraphs are separated by a blank paragraph in the middle so there are two line terminators "0D0A" between every two paragraphs if converted to hexadecimal. **To allow the length of data strands suitable for PCR amplification, we kept the number of symbols encoded in each data strand smaller than 32 (hypothetically there was no single word over 32 letters long). In order to keep the integrity of every word (in other words, one word should not be divided into different DNA strands, otherwise the searching would be difficult), we stopped the inclusion of words as we detected a whitespace symbol ("20" in hex) closest to the 32-letter limit. We also supplemented "20"s in hex to: 1) extend the data segment to be encoded in one data strand if the length was too short for sequencing and 2) make the length of data in one segment a multiple of 4 to support the pre-defined interval selection, which required at least 4 hexadecimal digits (16 bits) to make up one data unit.

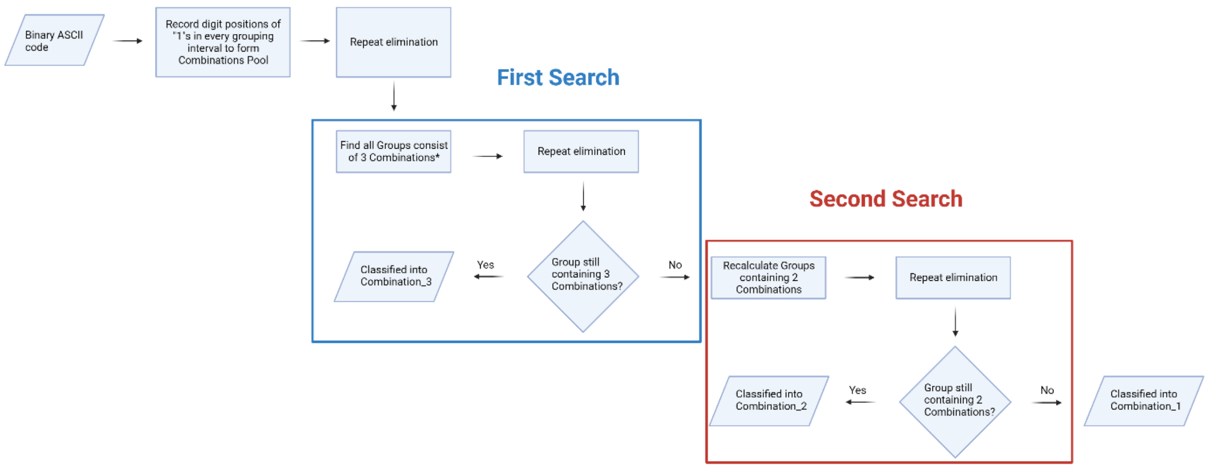

Supplementary Fig. 8. **Step 2: NCG algorithm to search groups without position collisions.** In general, a greedy algorithm was used in the group searching process of NCG coding. *The maximum size of a group is restricted to three, because oligos are made up of four different bases and there must be one available base to fill the non-occupied positions. Detailed explanations of the functions used in this algorithm can be found in the annotations of the source code. In the example of the 40 abstracts used in this study, the NCG algorithm produced 717 groups in total, among which 640 are classified into Combination_1, 76 are in Combination_2, and 1 is in Combination_3.

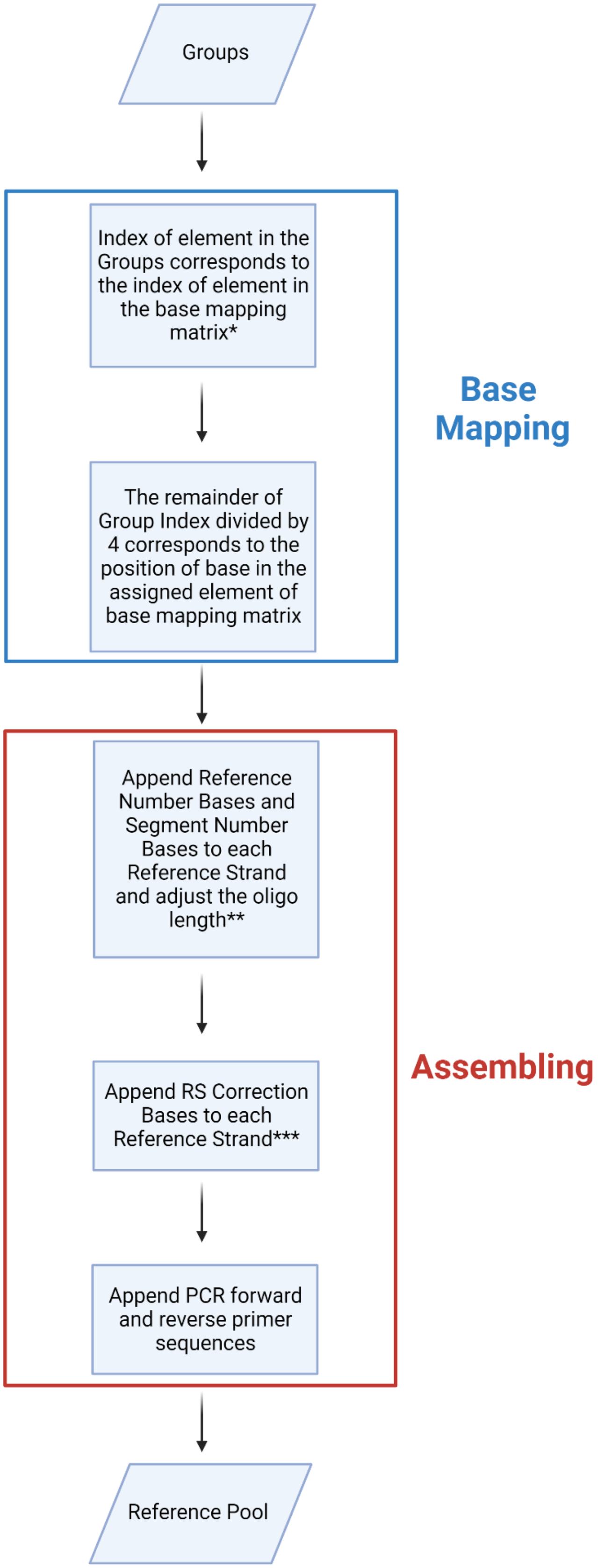
Groups
Index of element in the Groups corresponds to the index of element in the base mapping matrix*
The remainder of Group Index divided by 4 corresponds to the position of base in the assigned element of base mapping matrix
Base Mapping
Append Reference Number Bases and Segment Number Bases to each Reference Strand and adjust the oligo length**
Append RS Correction Bases to each Reference Strand***
Append PCR forward and reverse primer sequences
Assembling
Reference Pool

Supplementary Fig. 9. Step 3: Generation of reference strands. *The base mapping matrix in this study is defined as ["ACGT", "CGTA", "GTAC", "TACG"]. The aim to create a base mapping matrix is not only to provide an order for base mapping but also to avoid the appearance of base repeats and to balance GC content as much as possible. After inclusion of PCR forward and reverse primer target sequences, the generated sequence contains no $n > 3$ homopolymers and only contains 8 "AAA"s, 16 "CCC"s, 2 "GG"s, and 12 "TTT"s (taking up around 0.12%, 0.23%, 0.03%, and 0.18% of the total bases, respectively). The average GC content of all reference strands is 50.8%, fluctuating from 43.7% to 56.3% for each strand. **In this case, we selected 80 as the length of payload of a reference strand. As the total number of combinations was 717, the last oligo segment was composed of 77 bases. To make computation of RS code more convenient, we padded bases "ACT" at the end of the last segment so the length became 80 as well. ***We used reedsolo, a Python package available from GitHub, to produce RS code for the reference strands. In each oligo, The RS code was generated through the following process: 1) Convert the payload, including bases indicating reference and segment number, to double binary digits by the following mapping principle: ["A": "00", "C": "01", "G": "10", "T": "11"]; 2) Include every 8 binary digits in a list; 3) Compute RS code for the binary lists using reedsolo; 4) Convert the RS code to ternary; 5) Conduct a homopolymer-free base mapping[1] on ternary RS code to obtain RS oligo bases. We set two error correcting symbols, allowing us to correct one element in the binary list, which corresponds to at least one base in the reference strand. Given that the empirical error rate for Illumina-based sequencing is usually less than 1%[7], the ability to correct one base is enough if the payload is 80-nt long. After conversion, the number of RS correcting bases in each reference strand was 12. Every reference strand has a length of: L(Reference Strand) = 2L(primer targets) + L(Reference Number) + L(Segment Number) + L(Payload) + L(RS correction) = 2 × 21 + 4 + 4 + 80 + 12 = 142 nt. Detailed explanations of the functions used in this algorithm can be found in the annotations of the source code.

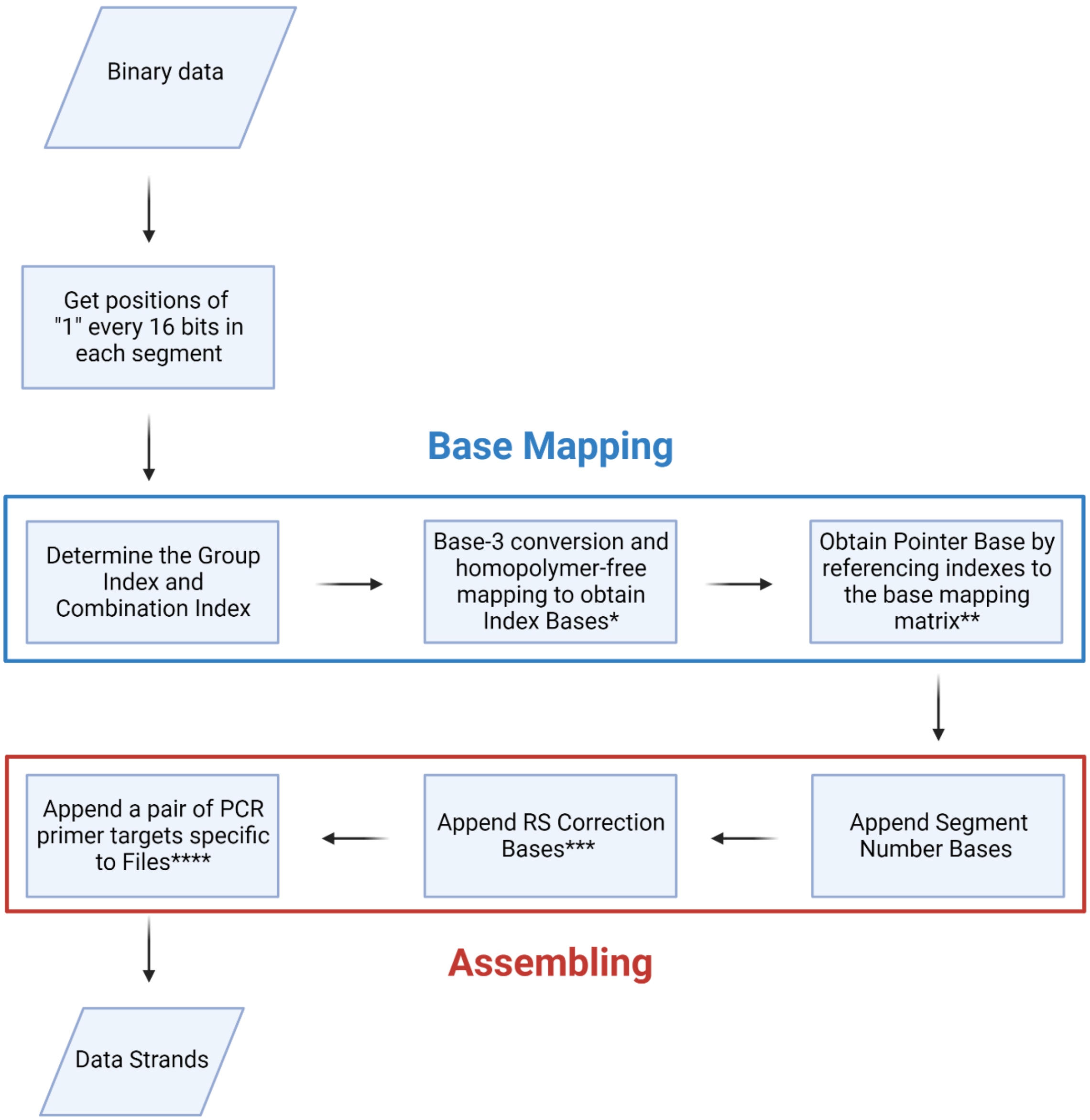

Supplementary Fig. 10. Step 4: Generation of data strands. *Base-3 conversion and homopolymer-free mapping[1] aims to avoid homopolymers in the data strand. After supplementing PCR forward and reverse primer target sequences, the generated sequence contains no $n > 3$ homopolymers and contains 25 "AAA"s, 0 "CCC"s, 10 "GGG"s, and 3 "TTT"s (taking up around 0.044%, 0%, 0.018% and 0.005% of the total bases, respectively). In addition, a balanced GC content was maintained among all data strands. The average GC content of all data strands was 51.5%, fluctuating from 43.6% to 60.4% for each strand. **The determination of the pointer base is a matching process based on the base mapping matrix mentioned in Step 3. The combination index matches the position of the base set of the base mapping matrix and the remainder of the group index divided by 4 matches to the position of the exact base in the base set. ***The

determination of the RS correction code is similar to what is described in Step 3, except that the length of the binary codes converted from the payload may not be a multiple of 8. To address this issue, we appended one more pointer base as the assistance base at the end of each data unit, so each data unit contains eight bases. The appended pointer bases are only applied to facilitate RS correction but are not actually synthesized so that the coding potential was not compromised. We set two error correcting symbols so that at least one base in the data strand can be corrected. Since the length of a data strand (excluding PCR primer targets) ranged 100–128, we believe two error correcting symbols are sufficient for the estimated 1% error rate in DNA sequencing. If PCR primer target sequences are included, the length of a data strand will be 142–170 nt. ****In this work, the data to be encoded include 40 abstracts of journal articles. To allow random access of an abstract in full text, each abstract was assigned a file number as its ID, which corresponded to a unique set of PCR forward and reverse primer target sequences added to every data strand belonging to this file. In the decoding process, the PCR primer targets can be directly mapped to file numbers.

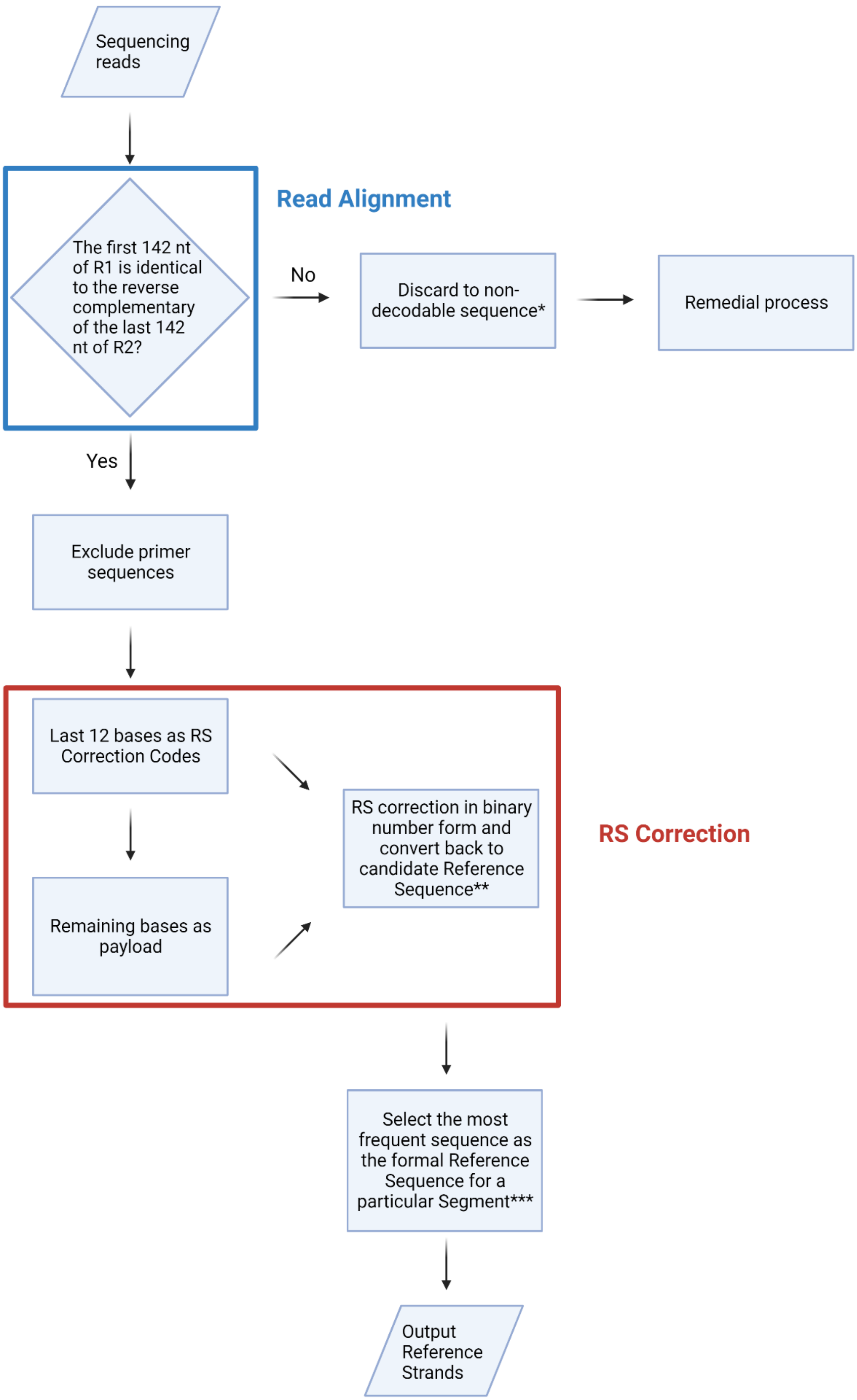
Sequencing reads
Read Alignment
The first 142 nt of R1 is identical to the reverse complementary of the last 142 nt of R2?
No
Discard to non-decodable sequence*
Remedial process
Yes
Exclude primer sequences
Last 12 bases as RS Correction Codes
Remaining bases as payload
RS correction in binary number form and convert back to candidate Reference Sequence**
RS Correction
Select the most frequent sequence as the formal Reference Sequence for a particular Segment***
Output Reference Strands

Supplementary Fig. 11. Step 5: Decoding the reference strands. *Due to the difficulty in base-calling cycles of elements of the base mapping matrix (e.g., "ACGTACGTACGT", "CGTACGTACGTA", etc.), some reads in which the first 142 bases in the R1 read did not match the reverse complementary of the last 142 bases in the R2 read may not be decodable. Some remedial procedures can be taken to recover those non-decodable sequences. The remedial algorithm is discussed in the next figure. **The RS code corrects binary numbers converted from the reference bases according to the mapping principle consistent with that in Supplementary Fig. 9: {"A": "00", "C": "01", "G": "10", "T": "11"}. To avoid homopolymers appearing in the RS correction bases, the calculated binary RS correction codes were converted to ternary, and then performed a homopolymer-free mapping[1] to obtain the sequence. We conducted the RS checking in a reverse process. ***The reference and segment numbers were obtained through a reversed mapping from homopolymer-free sequences to ternary numbers, and then the ternary numbers were converted to decimals.

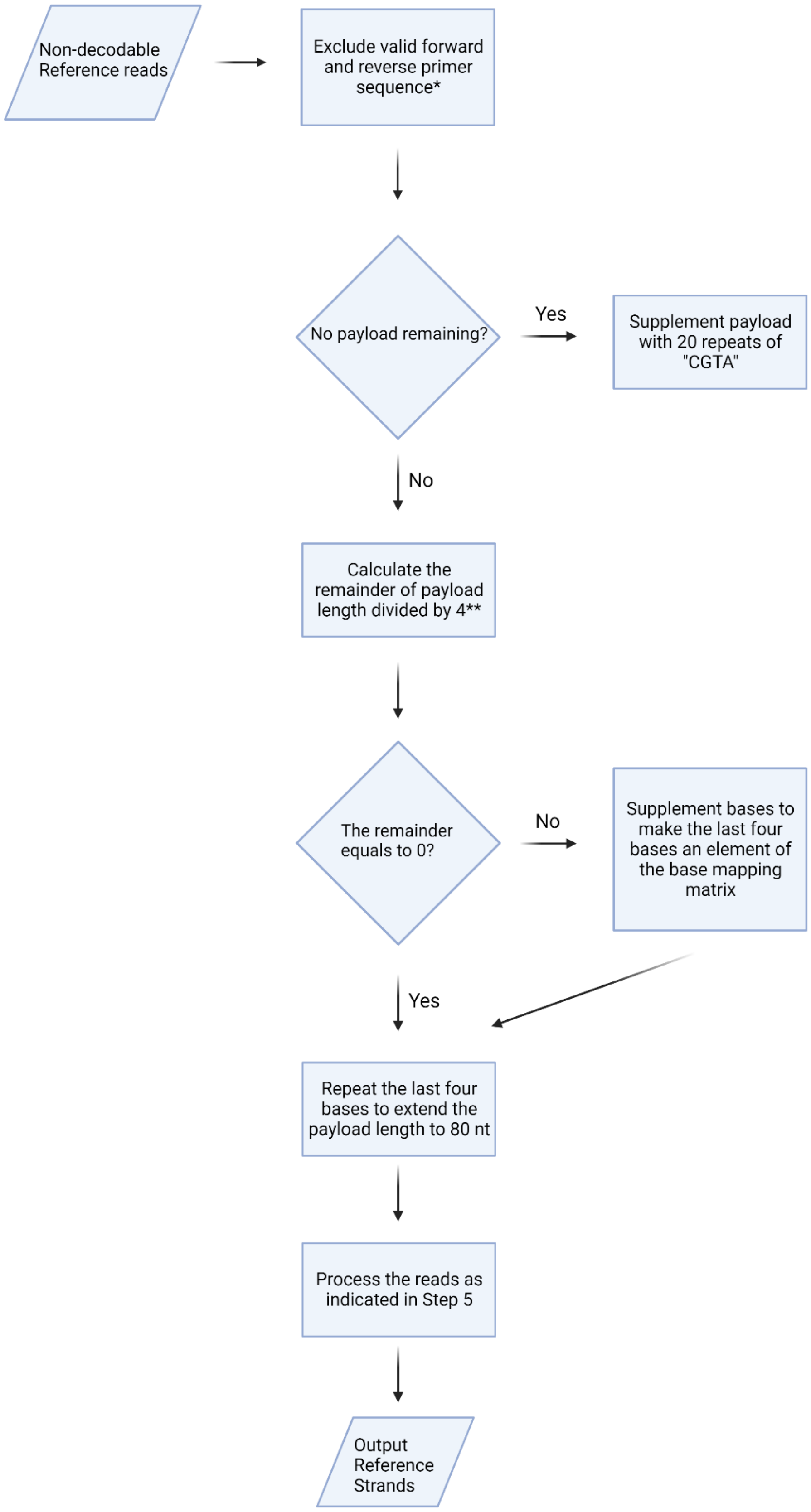
Non-decodable Reference reads
Exclude valid forward and reverse primer sequence*
No payload remaining?
Yes
Supplement payload with 20 repeats of "CGTA"
No
Calculate the remainder of payload length divided by 4**
The remainder equals to 0?
No
Supplement bases to make the last four bases an element of the base mapping matrix
Yes
Repeat the last four bases to extend the payload length to 80 nt
Process the reads as indicated in Step 5
Output Reference Strands

Supplementary Fig. 12 Step 5.1: Recover the sequences non-decodable in Step 5. Sequencing errors may occur when reading sequences comprising cycles of nucleobases, as mentioned in Supplementary Fig. 11, resulting in a loss of information in the dictionary and potentially impacting the decoding of data. However, through reference and segment numbers decoded from the reads, we found that every segment of the reference can be covered in sequencing, with 18 out of 144 (12.5%) strands incompletely read. This figure introduces a method to recover these partially read sequences. *The reason these sequences were non-decodable was mainly because the middle part of the sequences were missing. We observed that, in most cases, the read length was not reduced and some random sequence pieces were inserted after the PCR reverse primer target sequence. Therefore, the first step is to identify the position of the reverse primer target (if any) in each read and extract the sequence fragment between the forward and reverse primer targets. **Since all the non-decodable parts were cycles of elements of the base mapping matrix, our strategy was to supplement bases from the interrupted point of a read to make the length of a payload equal to its regular length, which is 80 nt. The addition of bases should depend on 1) the number of bases left to complete an element of the base mapping matrix and 2) the remaining bases in the last element of the base mapping matrix. A detailed base supplementing rule can be found in the source code. ***We found it is necessary to count and pick the most frequently decoded sequence in this step because sometimes the bases encoding the reference or segment number might be wrongly identified in a corrupted read, and the RS code failed in correcting this kind of error so this read would be distributed to a wrong reference or segment position. Therefore, we could not simply claim the reference and segment position immediately after one successfully decoded sequence was observed. Instead, it is necessary to gather all possible sequences and choose the most likely one according to their frequency of appearance.

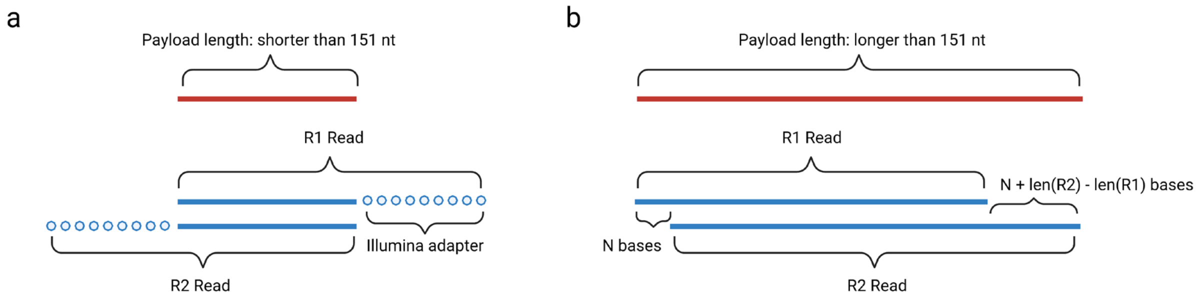

Supplementary Fig. 13. Read alignment for different payload length conditions. a, When the payload length is fewer than 151 nt (to be exact, 142 nt or 149 nt), the payload is fully covered by a single read and the rest of the bases are a portion of the Illumina adapters. We need to double-check the consistency of base calling of R1 and the reverse complementary of R2 to confirm this sequence. **b,** When the payload length is more than 151 nt (to be exact, 156 nt, 163 nt, or 170 nt), a single read does not cover all bases in the payload and we need to detect the maximal overlap of the R1 read and the R2 read. Since the sequence of the R1 read is the standard for decoding, bases missing in the R1 read should be supplemented. In practice, the read length of R1 and R2 may not be equal, and we denote the difference in length as len(R2) – len(R1). The number of bases to be supplemented to R1 can therefore be expressed by N + len(R2) – len(R1), where N marks the position of the starting base of R1 where the following bases overlap with the reverse complementary of R2. If such overlap is detected, the complete payload is assumed to be the sequence of R1 plus the last N + len(R2) – len(R1) bases of the reverse complementary of R2.

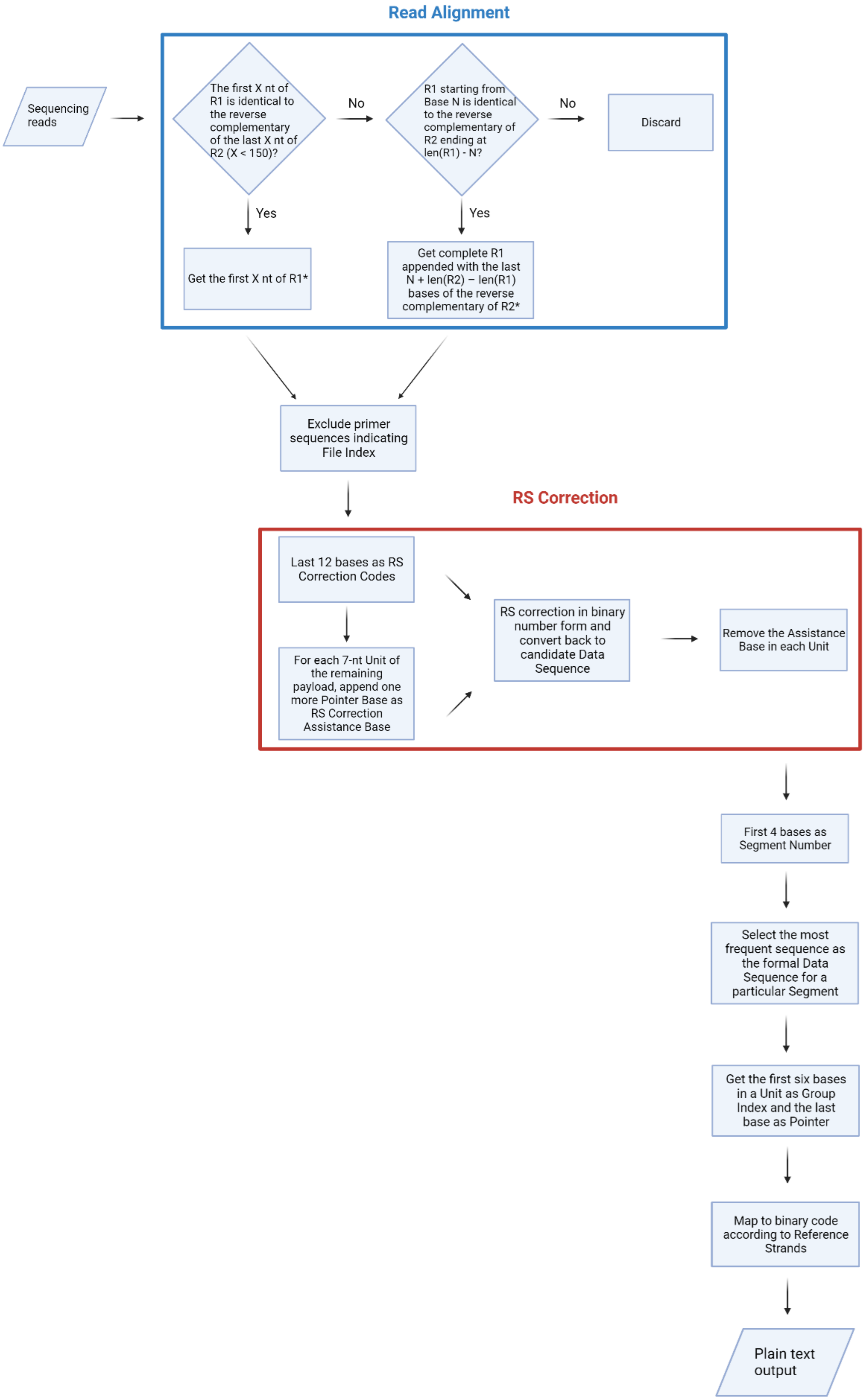

Read Alignment
Sequencing reads
The first X nt of R1 is identical to the reverse complementary of the last X nt of R2 (X < 150)?
No
R1 starting from Base N is identical to the reverse complementary of R2 ending at len(R1) - N?
No
Discard
Yes
Yes
Get the first X nt of R1*
Get complete R1 appended with the last N + len(R2) – len(R1) bases of the reverse complementary of R2*
Exclude primer sequences indicating File Index
RS Correction
Last 12 bases as RS Correction Codes
For each 7-nt Unit of the remaining payload, append one more Pointer Base as RS Correction Assistance Base
RS correction in binary number form and convert back to candidate Data Sequence
Remove the Assistance Base in each Unit
First 4 bases as Segment Number
Select the most frequent sequence as the formal Data Sequence for a particular Segment
Get the first six bases in a Unit as Group Index and the last base as Pointer
Map to binary code according to Reference Strands
Plain text output

Supplementary Fig. 14. Step 6: Decoding the data strands. Decoding the data strands requires a read alignment of R1 and the reverse complementary of R2 (see Supplementary Fig. 13) to ensure that the sequences gathered from R1 and R2 are consistent. The remaining procedure of data strand decoding is a reverse process of data strand generation, which is shown in Supplementary Fig. 10. *The basis for considering these conditions in sequence processing is elaborated in Supplementary Fig. 13.

4. Supplementary Experimental Figures

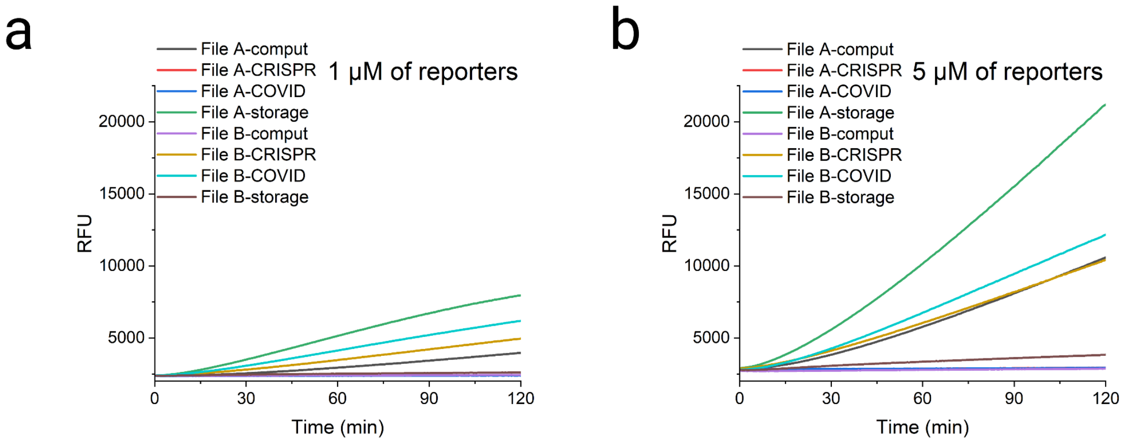

Supplementary Fig. 15. Concentration optimization of ssDNA reporters used in SEEKER for DNA data storage. a 1 μM of reporters. **b** 5 μM of reporters. A higher concentration of reporters produced a stronger fluorescence response and a clearer distinguishment between target groups (files containing the keyword) and non-target groups (files not containing the keyword). Source data are provided as a Source Data file.

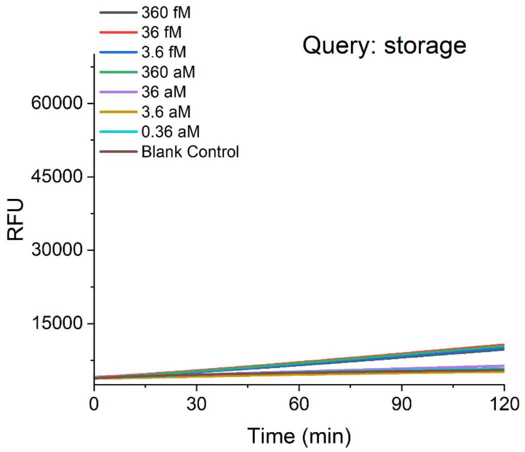

Supplementary Fig. 16. SEEKER without PCR amplification. This figure shows the case where the query was "storage" and the sample being searched was the entire oligo pool encoding 40 abstracts of journal articles without PCR-based random access to a specific file. The concentration of the 1× oligo pool was 360 fM. If no pre-amplification is implemented, CRISPR-based searching would not produce any significant signals for either the original or the diluted oligo pool. Although the RFU values were higher for samples at 360 aM or higher concentration, that difference was not as significant as the pre-amplified samples as shown in Fig. 3e in the main text. A weak fluorescence response for the non-amplified oligo pool at a picomolar concentration level allows us to combine SEEKER with PCR-based random access, otherwise the strong background fluorescence interferences may lead to massive misrecognition of target files. Source data are provided as a Source Data file.

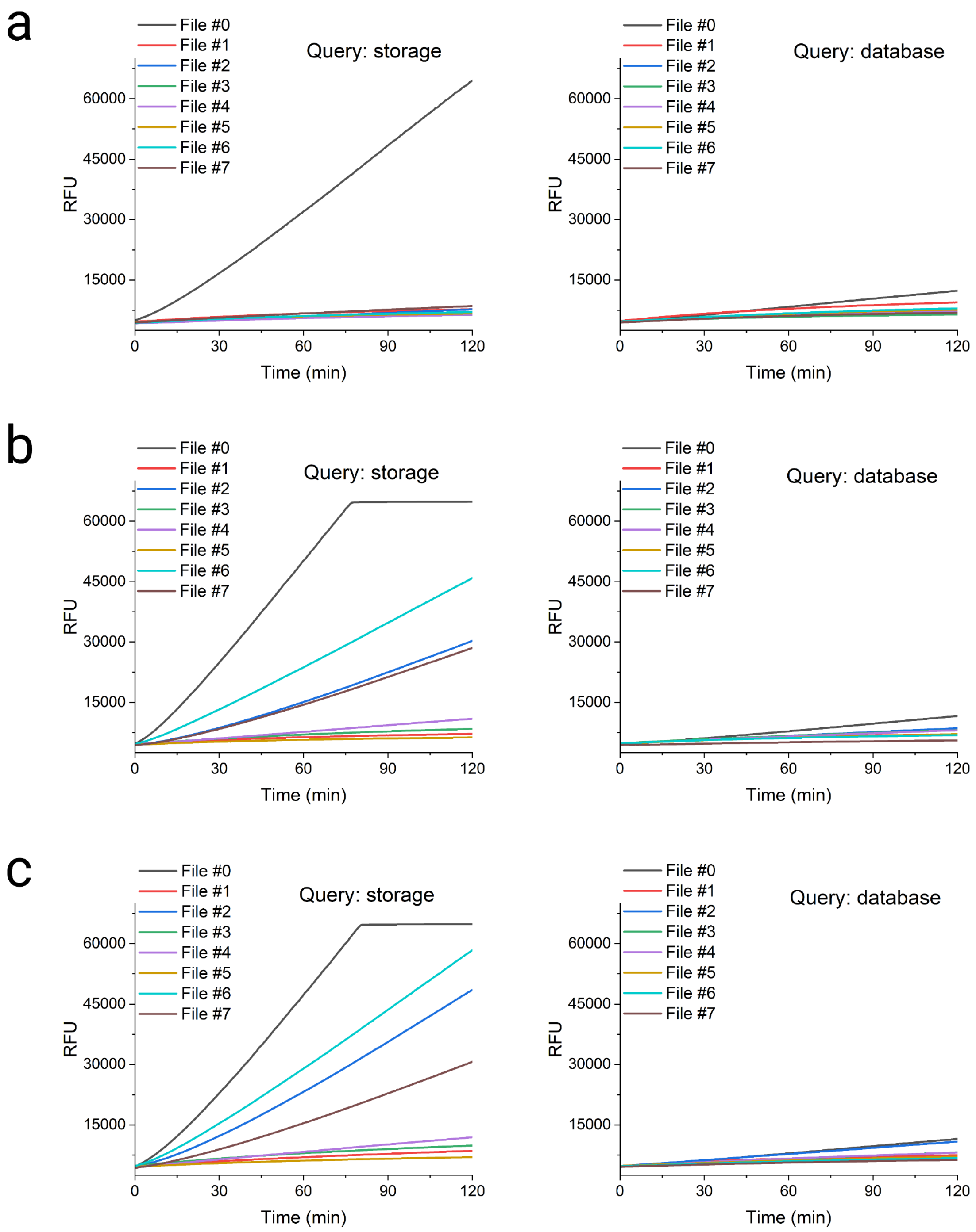

Supplementary Fig. 17. Optimization of template concentration for SEEKER on amplified oligo pool. The final concentration of the oligo pool was **a** 360 aM, **b** 3.6 fM, or **c** 36 fM and Files #0–#7 were accessed through PCR. The graphs show real-time fluorescence responses when using SEEKER to search the keywords "storage" and "database". In Files #0–#7, the target files containing the keyword "storage" were

File #0, #2, #6, and #7, while none of the files contained the keyword "database". When the concentration of the oligo pool was 360 aM, only File #0, which contained the most repeats of the keyword "storage" had a strong response. This indicates a template concentration as low as 360 aM was not enough for SEEKER to distinguish target files containing few repeats of keywords. When the final concentration of the oligo pool raised to 3.6 fM and 36 fM, all the target files were correctly identified, and a higher endpoint fluorescence was found for Files #2 and #6 at a higher template concentration. We refrained from testing higher concentrations as the amplification can be suppressed at high template concentrations, as suggested by the results in Fig. 3e in the main text. Importantly, as the template concentration increased to 36 fM, the fluorescence responses for non-target files remained low and significantly weaker than those for target files, which enlarged the RFU gap between target and non-target files and made the distinguishment much clearer. The optimal template concentration was therefore determined to be 36 fM. Source data are provided as a Source Data file.

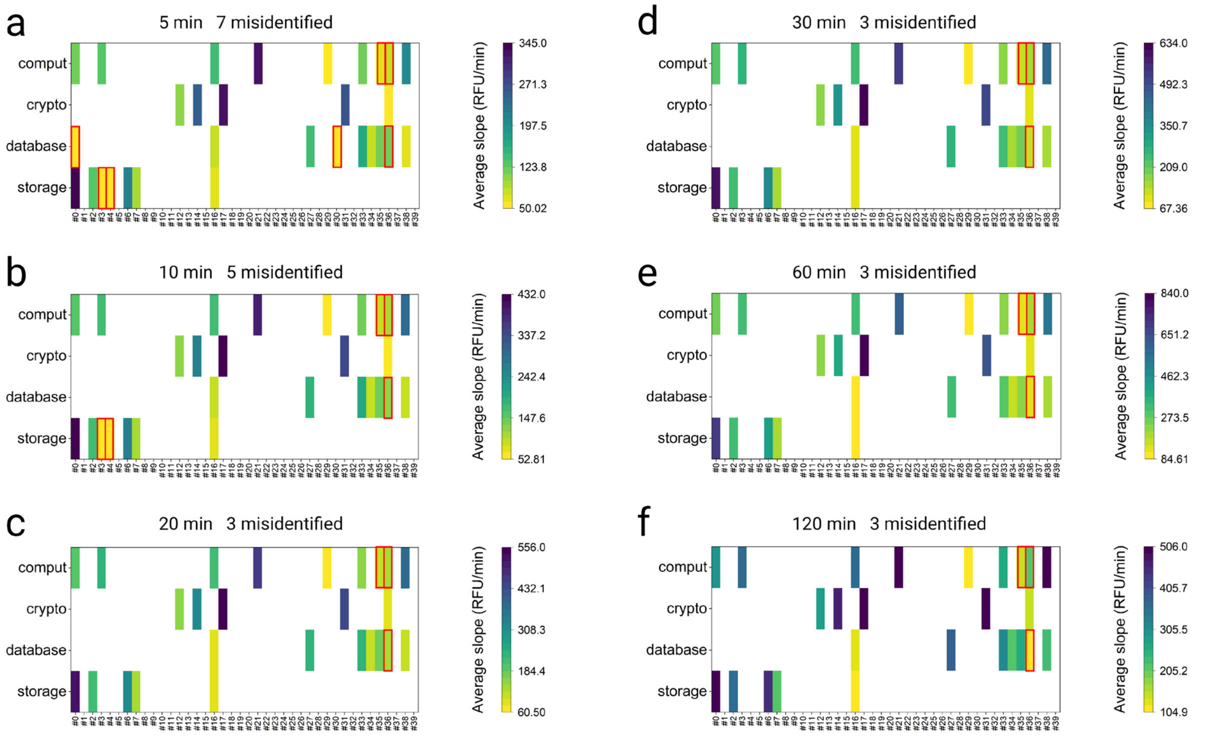

Supplementary Fig. 18. Misidentification by SEEKER at different reaction times. The slope of the fluorescence was used to identify whether the file being searched was the "target file" containing a particular keyword. Here, the slope was the average fluorescence change per minute during the reaction period. The recording time points were **a** 5 min; **b** 10 min; **c** 20 min; **d** 30 min; **e** 60 min; **f** 120 min. The threshold was set as the minimum slope of a "target file" when being searched by the four queries shown in the figures. The files that did not actually contain the keyword, but had a slope higher than the threshold, are considered to be misidentified files and are marked by red boxes. As the recording time prolonged from 5 min to 20 min, fewer files were misidentified. After 20 min, the number of misidentified files remained stable, which suggests that 20 min is the ideal recording time to obtain the lowest error rate in SEEKER. Among the 160 searches, 3 files were misidentified and the error rate was 3/160 = 1.875%. Source data are provided as a Source Data file.

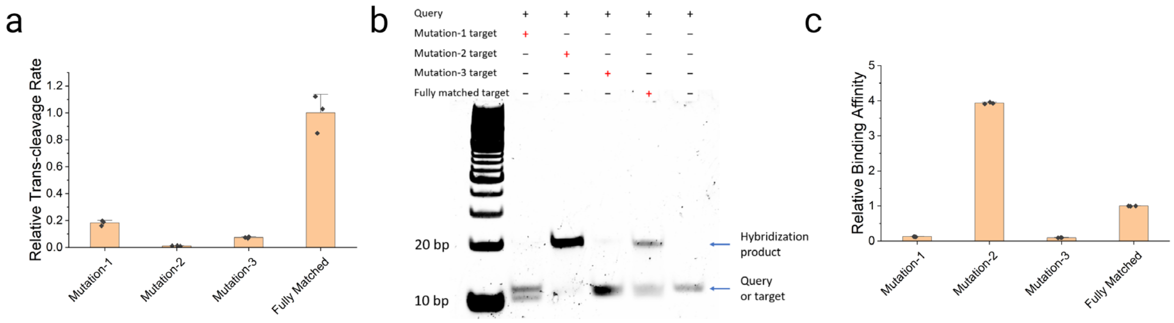

Supplementary Fig. 19. Comparison of specificity in sequence identification between SEEKER and the hybridization-based approach. a, The normalized trans-cleavage rate for mismatched and fully matched targets in SEEKER ($n = 3$. Data are presented as mean ± SD of three technical replicates). The average trans-cleavage rate for three experimental repeats for the fully matched target was normalized to 1.0. The results indicate that all mismatched targets had a trans-cleavage rate significantly lower than the fully matched one, with the highest among them reaching a value of 0.2. **b,** Gel electrophoresis showing the binding affinity between the query and targets. The band indicating hybridization between mutation-2 and the query was even darker than that for the reaction between the fully matched target and the query. **c,** Binding affinity revealed by measuring the band intensity ($n = 3$. Data are presented as mean ± SD of three independent measurements of the band intensity). The band intensity for mutation-2 was almost four times the intensity for the fully matched target, suggesting a possibly much stronger binding affinity between the query and a mismatched target. The list of oligo sequences used in this experiment can be found in Supplementary Table 6. Source data are provided as a Source Data file.

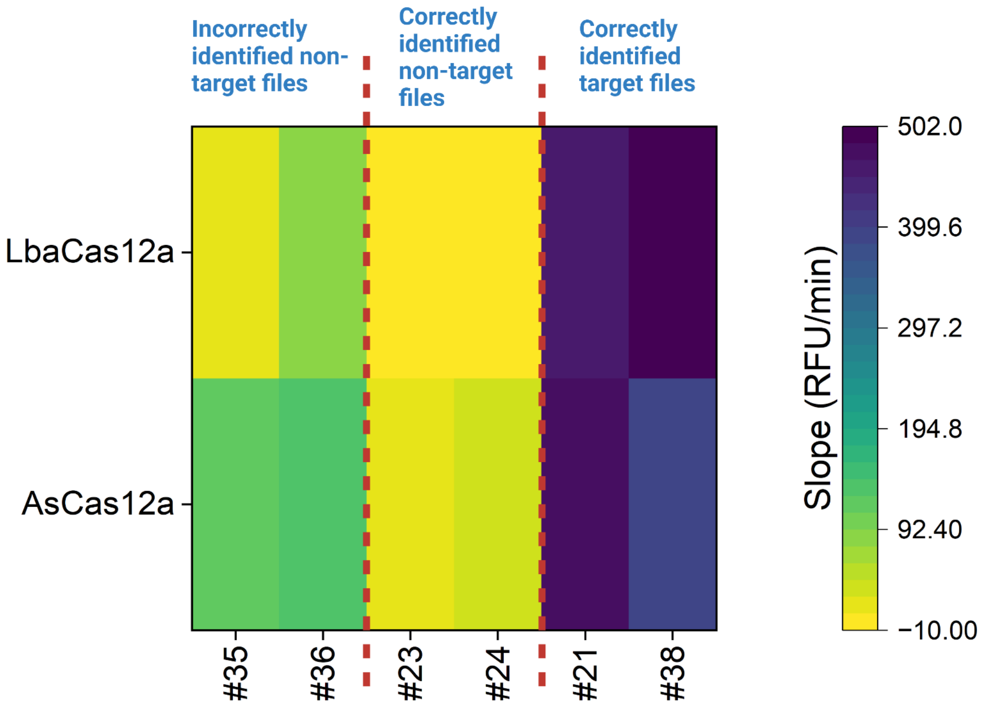

Supplementary Fig. 20. SEEKER using different enzymes. From Fig. 3i in the main text, the misidentification mostly occurred when the keyword "comput" was used as the query. We searched this keyword on correctly identified target files (File #21, File #38), correctly identified non-target files (File #23, File #24), and incorrectly identified non-target files (File #35, File #36) using another Cas12a ortholog EnGen® Lba Cas12a from NEB, and compared the results with AsCas12a. The figure shows the slopes of fluorescence at 20 min of experiments. LbaCas12a had a lower slope of fluorescence intensity for correctly identified non-target files while maintaining the same level of fluorescence for correctly identified target files. More importantly, LbaCas12a exhibited a weaker fluorescence response for incorrectly identified non-target files, particularly for File #35. These results suggest that LbaCas12a may perform better in distinguishing between target and non-target files, and the error rate may be further reduced if using LbaCas12a. Source data are provided as a Source Data file.

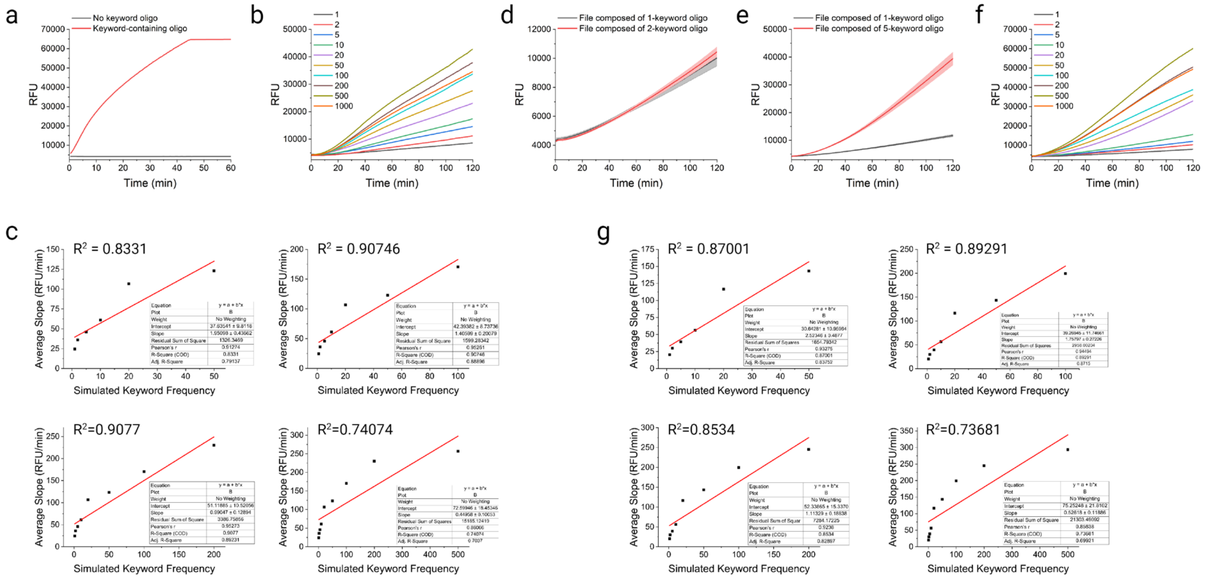

Supplementary Fig. 21. SEEKER with high keyword frequencies. a, Real-time fluorescence of CRISPR reactions with unamplified 12 nM of no-keyword and 1-keyword oligos, which confirmed that no-keyword oligos induced no interference in fluorescence response. **b**, Real-time fluorescence with keyword frequencies simulated with 1-keyword oligos. A higher keyword frequency typically corresponded to a faster enhancement in fluorescence intensity, while for a frequency of 1,000 the fluorescence response was slightly weaker. **c**, Linear fitting between average slopes and simulated keyword frequencies using 1-keyword oligos when keyword frequency was below 50, 100, 200 and 500. The result suggests that a maximum keyword frequency of 200 may still lead to a tolerable linear relationship between fluorescence enhancement rate and keyword frequency, but with even higher keyword frequencies the linearity might be significantly impaired. **d**, The comparison of fluorescence responses between amplified files containing merely 1-keyword oligos and merely 2-keyword oligos. There was no apparent variation in fluorescence kinetics between these two conditions. **e**, The comparison of fluorescence responses between amplified files containing merely 1-keyword oligos and merely 5-keyword oligos. The fluorescence response for file composed of merely 5-keyword oligos was significantly stronger than that for file composed of merely 1-keyword oligos. For **d** and **e**, experiments at each concentration were run in triplicate (n = 3) and graphs represent mean (bold line) ± SD (fill). **f**, Real-time fluorescence with keyword frequencies simulated with a mixture of 1-keyword, 2-keyword and 5-keyword oligos. Similar to the results with keyword frequencies simulated with 1-keyword oligos, as the keyword frequency increased, the fluorescence response became stronger accordingly. When keyword frequency reached 1,000, the fluorescence response slightly weakened. **g**, Linear fitting between average slopes and simulated keyword frequencies using multi-keyword oligos when keyword frequency was below 50, 100, 200 and 500. Similar to the condition when

merely using 1-keyword oligos, a relatively better linear relationship between fluorescence enhancement rate and keyword frequency was obtained when keyword frequency was not exceeding 200. The oligo sequences used in this experiment is listed in Supplementary Table 7. The distribution of 1-keyword, 2-keyword and 5-keyword oligos for each simulated keyword frequency in the mixed experimental setting is listed in Supplementary Table 8. The Source data are provided as a Source Data file.

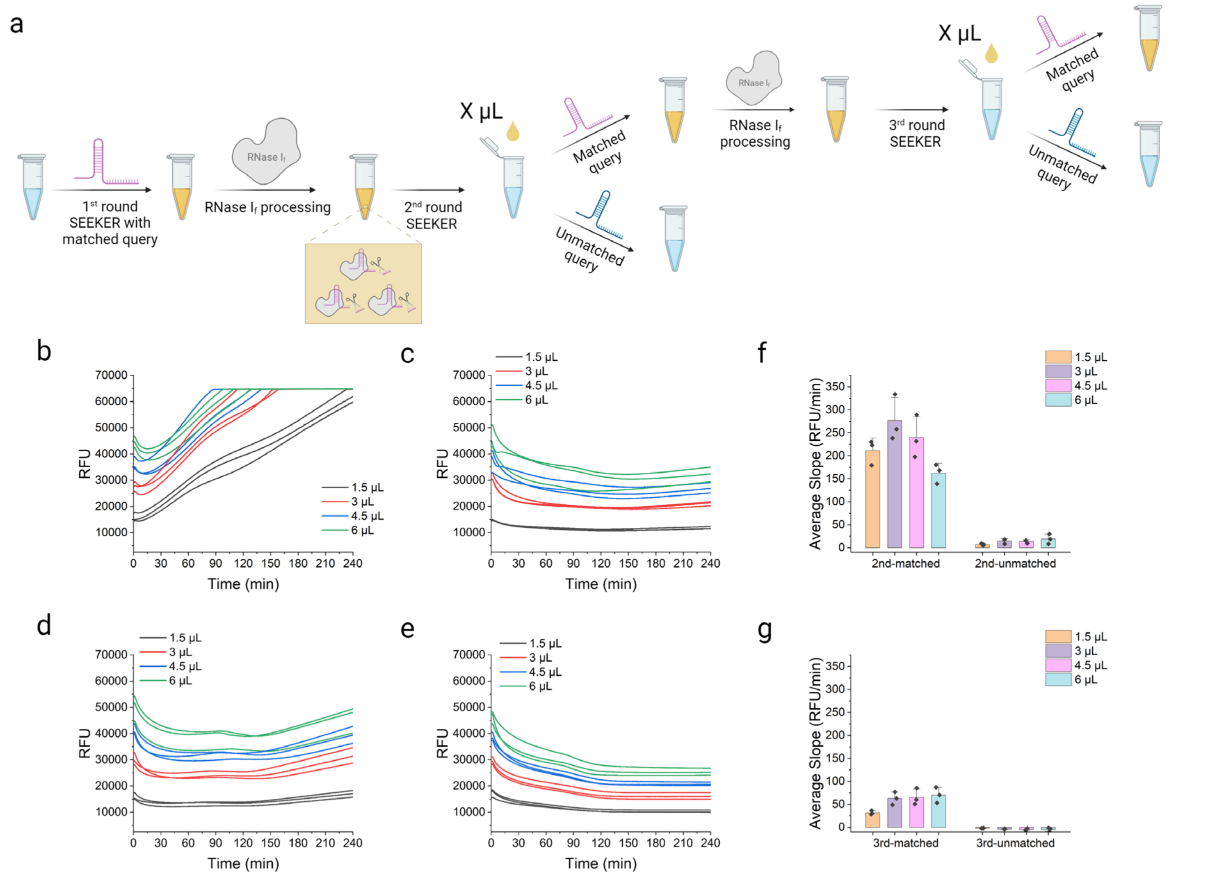

Supplementary Fig. 22. Reusable SEEKER. a, The scheme of experimental design of reusable SEEKER. In the experiment, we selectively amplified File #33 from the oligo pool we used for the molecular search in this work. First, we used the query "comput" to search for content in File #33, and the fluorescence was expected to saturate. Next, we treated the sample with RNase I_f at 37°C for 15 min followed by heat inactivation at 85°C for 30 min to remove the previous query crRNA. For the next round of the SEEKER search, we used the query "database" which was present in File #33, and the query "storage" which was absent. Different sample volumes ranging from 1.5 μL to 6 μL were added to the SEEKER reaction to investigate the impact of the amplicon sample volume on the reaction performance. Depending on the performance, more rounds of reusable SEEKER can be conducted. **b,** Real-time fluorescence of second-round SEEKER with a matched query. **c,** Real-time fluorescence of second-round SEEKER with an unmatched query. In Figs. **b-c**, the fluorescence intensity started to increase at about 30 min with the matched query, but continued to decay with an unmatched query, with only a slight increase after 120 min of reaction. **d,** Real-time fluorescence of third-round SEEKER with a matched query. **e,** Real-time fluorescence of third-round SEEKER with an unmatched query. In Figs. **d-e**, there was no obvious fluorescence enhancement in the first 120 min of reaction with both queries. However, in the reaction with

matched queries, a measurable fluorescence increase was observed after 120 min, while no apparent increase was found with an unmatched query. **f,** Slope of fluorescence for the second-round SEEKER with different sample volumes from the last reaction (*n* = 3. Data are presented as mean ± SD of three technical replicates). The fluorescence enhancement in the first 90 min was considered. **g,** Slope of fluorescence for the third-round SEEKER with different sample volumes from the last reaction (*n* = 3. Data are presented as mean ± SD of three technical replicates). The fluorescence enhancement in the last 120 min was considered. Source data are provided as a Source Data file.

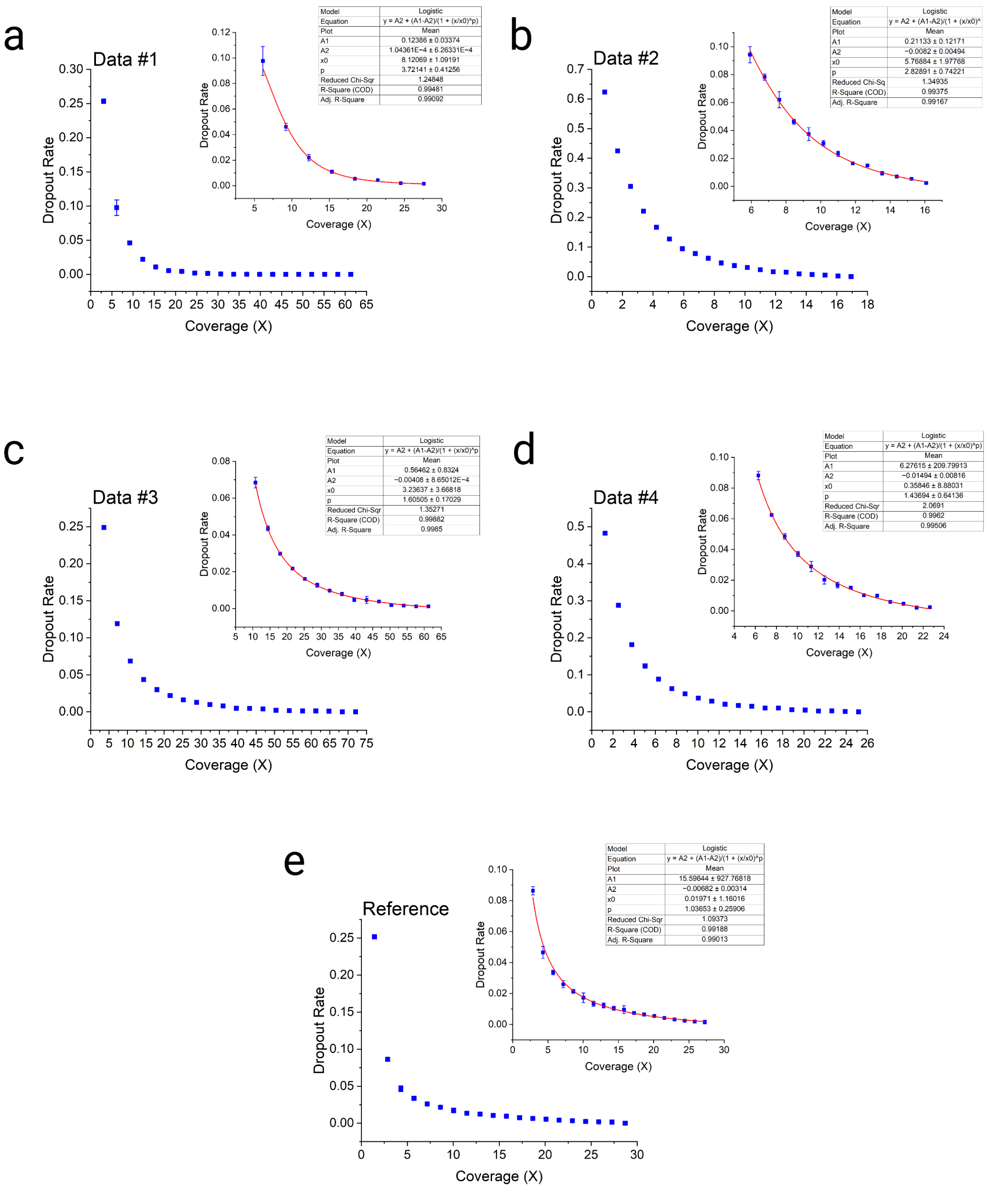

Supplementary Fig. 23. Dropout rates of subsampled reads in the original FASTQ files. In subsampling experiments, the reads were randomly selected, and the sequencing coverages dropped from 100% to 5% of the original coverages by 5% in each subsampling experiment. The dropout rate was defined as the proportion of oligo strands not captured at the specified sequencing coverage. Each dropout rate was the average of results from 20 independent subsamplings, which was repeated three times to eliminate the

bias in random read selection. Data are presented as mean ± SD. The amplicon samples were **a,** data amplicon pool #1 with files containing the word "storage" (File #0, #2, #6, #7, #16 included). **b,** data amplicon pool #2 with files containing the word "database" (File #16, #27, #33, #34, #35, #38 included). **c,** data amplicon pool #3 with files containing the word "crypto" (File #12, #14, #17, #31, #36 included). **d,** data amplicon pool #4 with files containing the word "comput" (File #0, #3, #16, #21, #29, #33, #38 included). **e,** reference amplicon pool (all reference strands included). Based on logistic regressions of sequencing coverage and the dropout rate, the minimum coverages to achieve a dropout rate less than 1% are 15.65×, 13.48×, 31.93×, 16.78×, and 14.35× for Data #1, Data #2, Data #3, Data #4, and Reference pool, respectively. Source data are provided as a Source Data file.

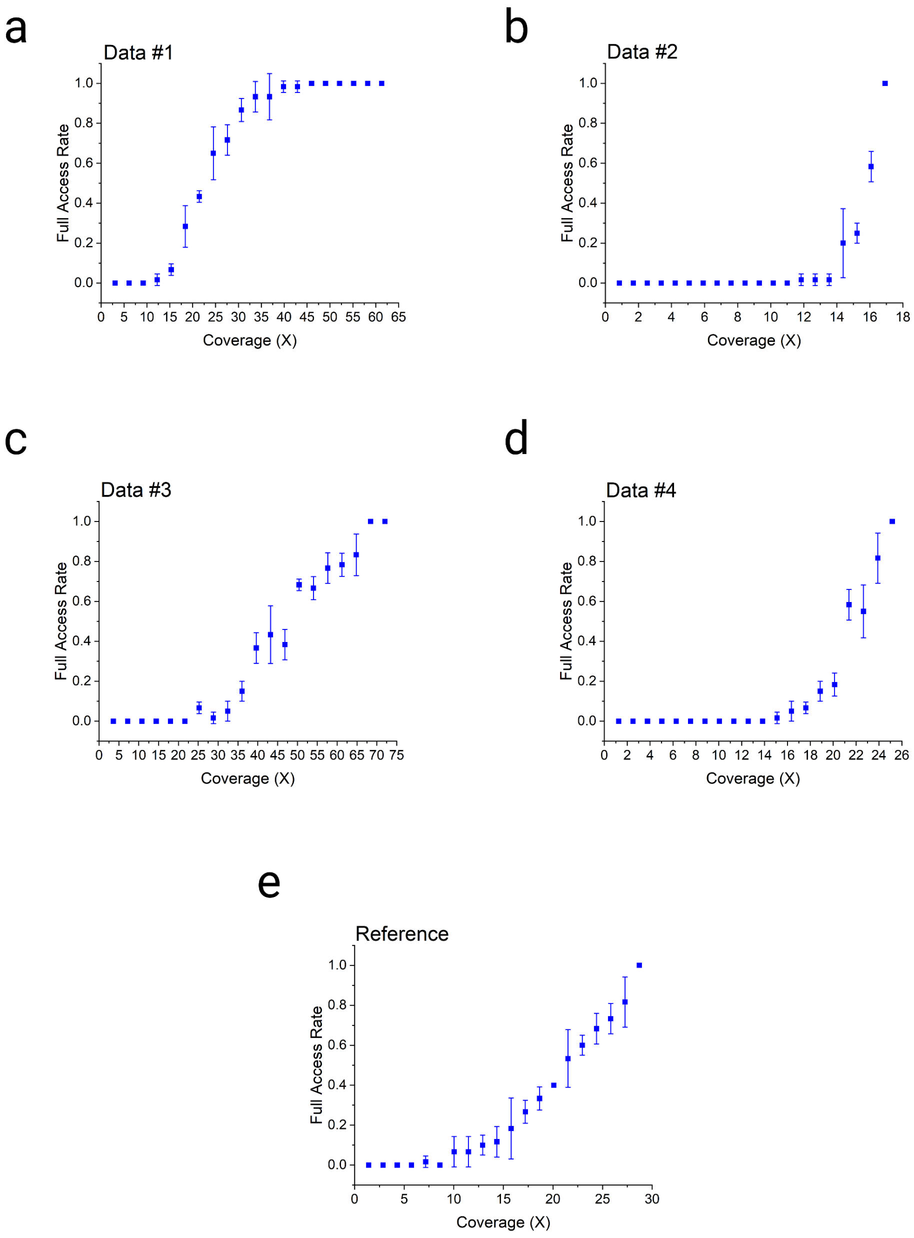

Supplementary Fig. 24. Full access rates of subsampled reads in the original FASTQ files. The full access rate is defined as the percentage of full access (zero dropouts) to amplicons in the sample. Each dropout rate was the average of results from 20 independent subsamplings, which was repeated three times to eliminate the bias in random read selection. Data are presented as mean ± SD. The samples used were the same as those in Supplementary Fig. 23 and are specified respectively in **a–e**. A full access rate of 1.0 means all the 20 randomly selected subsamples of the original sample can fully cover all the amplicon sequences that need to be decoded. We observed that only data #1 reached a stable full access rate at 1.0

within its original coverage. Therefore, the original coverages might be too low to determine the minimum coverage required to securely decode all data in an amplicon pool. One statistical method to address this issue is to expand the sample size by resampling, which is explained in Figures 5c and 5d in the main text. Source data are provided as a Source Data file.

5. Supplementary Tables

Sample	Number of reads	Number of amplicons to be read	Sequencing coverage	% of decoded
Data #1	13,487	220	61.30X	100%
Data #2	3,842	227	16.93X	100%
Data #3	14,121	196	72.05X	100%
Data #4	7,069	281	25.16X	100%
Reference	4,133	144	28.70X	100%
Total	42,652	1,068	39.94X	100%

Supplementary Table 3. General sequencing statistics. The sequencing coverage is derived by dividing the total number of reads by the number of amplicons to be read. All the amplicons to be read can be recovered from the sequencing reads.

Reference	Segment	Sequence excluding primer, index and RS code (5’-3’)
#0	#0	CGTACGTACGTACGTACGTACGTACGTACGTACGTACGTACGTACGTACGTACGTACGTACGTACGTACGTACGTACGTA
#0	#1	CGTACGTACGTACGTACGTACGTACGTACGTACGTACGTACGTACGTACGTACGTACGTACGTACGTACGTACGTACGTA
#0	#2	CGTACGTACGTACGTACGTACGTACGTACGTACGTACGTACGTACGTACGTACGTACGTACGTACGTACGTACGTACGTA
#0	#3	CGTACGTACGTACGTACGTACGTACGTACGTACGTACGTACGTACGTACGTACGTACGTACGTACGTACGTACGTACGTA
#0	#4	CGTACGTACGTACGTACGTACGTACGTACGTACGTACGTACGTACGTACGTACGTACGTACGTACGTACGTACGTACGTA
#0	#6	CGTACGTACGTACGTACGTACGTACGTACGTACGTACGTACGTACGTACGTACGTACGTACGTACGTACGTACGTACGTA
#0	#7	CGTACGTACGTACGTACGTACGTACGTACGTACGTACGTACGTACGTACGTACGTACGTACGTACGTACGTACGTACGTA
#2	#0	CCGTACGTACGTACGTACGTACGTACGTACGTACGTACGTACGTACGTACGTACGTACGTACGTACGTACGTACGTACGT
#8	#0	CGTACGTACGTACGTACGTACGTACGTACGTACGTACGTACGTACGTACGTACGTACGTACGTACGTACGTACGTACGTA
#8	#1	CGTACGTACGTACGTACGTACGTACGTACGTACGTACGTACGTACGTACGTACGTACGTACGTACGTACGTACGTACGTA
#8	#2	CGTACGTACGTACGTACGTACGTACGTACGTACGTACGTACGTACGTACGTACGTACGTACGTACGTACGTACGTACGTA
#8	#3	CGTACGTACGTACGTACGTACGTACGTACGTACGTACGTACGTACGTACGTACGTACGTACGTACGTACGTACGTACGTA
#8	#4	CGTACGTACGTACGTACGTACGTACGTACGTACGTACGTACGTACGTACGTACGTACGTACGTACGTACGTACGTACGTA
#8	#6	CGTACGTACGTACGTACGTACGTACGTACGTACGTACGTACGTACGTACGTACGTACGTACGTACGTACGTACGTACGTA
#8	#7	CGTACGTACGTACGTACGTACGTACGTACGTACGTACGTACGTACGTACGTACGTACGTACGTACGTACGTACGTACGTA
#10	#0	ACGTACGTACGTACGTACGTACGTACGTACGTACGTACGTACGTACGTACGTACGTACGTACGTACGTACGTACGTACGT
#10	#1	ACGTACGTACGTACGTACGTACGTACGTACGTACGTACGTACGTACGTACGTACGTACGTACGTACGTACGTACGTACGT
#10	#2	ACGTACGTACGTACGTACGTACGTACGTACGTACGTACGTACGTACGTACGTACGTACGTACGTACGTACGTACGTACGT

Supplementary Table 4. List of missing reference strands before the remedial process.

Sample	Total reads	Total valid reads	Matched reads before RS correction	Matched reads ratio	Matched reads after RS correction	Matched reads ratio	Improved matched reads ratio by RS correction
Data #1	13,487	12,309	8,422	68.42%	9,138	74.24%	5.82%
Data #2	3,842	2,778	2,032	73.15%	2,213	79.66%	6.51%
Data #3	14,121	12,736	9,693	76.11%	10,425	81.85%	5.74%
Data #4	7,069	6,742	4,871	72.25%	5,251	77.88%	5.63%
Reference	4,133	4,056	3,396	83.73%	3,543	87.35%	3.62%
Total	42,652	38,621	28,414	73.57%	30,570	79.15%	5.58%

Supplementary Table 5. Detailed sequencing statistics. In this table, "total reads" refers to the total number of reads in the raw FASTQ file before any processing. "Total valid reads" refers to the number of remaining reads after read alignment. "Matched reads before RS correction" refers to the number of aligned sequences identical to one of the data or reference strands before RS correction. "Matched reads after RS correction" refers to the number of aligned sequences identical to one of the data or reference strands after RS correction. On average, RS correction improves the ratio of matched reads to total reads by 5.58%.

Name	Sequence (5'-3')
crRNA-storage_1	UAAUUUCUACUCUUGUAGAUUAGCGUCACGCGUCGCUCGUC
Fully matched DNA target	GACGAGCGACGCGTGACGCTA
Mutation_1	GACGACGCTCGCGTGACGCTA
Mutation_2	GACGAGCGAGCGCTGACGCTA
Mutation_3	GACGAGCGACGCGACTGGCTA

Supplementary Table 6. Oligo sequences for comparison of specificity between SEEKER and the conventional hybridization approach (red bases refer to mismatches).

Name	Sequence (5'-3')
**crRNA-storage_1**	UAAUUUCUACUCUUGUAGAUUAGCGUCACGCGUCGCUCGUC
**No keyword oligo**	TGTCTTCTCCAGCAACGAATGGTACTGTACTGGTAGATGGCGCACGGCGACGTGTCTATGGTGATACGCTACATGAGTCACGCTCGCAATGAGCAGTAGTCGGTCGCGCGTCGCAGTAGTGGCCAACAATCCTACTGCCTAG
**1-keyword oligo**	TGTCTTCTCCAGCAACGAATGGTCTGACGAGCGACGCGTGACGCTAGCTCGTGATATCGCGTCGTCAGACAGTGGAGTATGATGAGCAGTATCATGACACGTGTCTGCAGATATGTGTGTAGTAGCACTAGCTATCGAGTACGCCAACAATCCTACTGCCTAG
**2-keyword oligo**	TGTCTTCTCCAGCAACGAATGGTCTGACGAGCGACGCGTGACGCTAGCTCGTGATATCGCGTCGTCAGACAGTGGAGTATGATGAGCAGTATCATGACACGTGTCTGCAGACGAGCGACGCGTGACGCTAGCTATCGAGTACGCCAACAATCCTACTGCCTAG
**5-keyword oligo**	TGTCTTCTCCAGCAACGAATGGTCTGACGAGCGACGCGTGACGCTAGACGAGCGACGCGTGACGCTAGACGAGCGACGCGTGACGCTAGACGAGCGACGCGTGACGCTAGACGAGCGACGCGTGACGCTAGCTATCGAGTACGCCAACAATCCTACTGCCTAG
**Forward primer**	TGTCTTCTCCAGCAACGAATG
**Reverse primer**	CTAGGCAGTAGGATTGTTGGC

Supplementary Table 7. Oligo sequences for the experiment of higher keyword frequency simulation (primer targets are marked in purple and keyword sequences are marked in orange).

Total keyword frequency	Keyword frequency contributed by		
	1-keyword oligos	2-keyword oligos	5-keyword oligos
**1**	1	0	0
**2**	2	0	0
**5**	5	0	0
**10**	8	2	0
**20**	16	4	0
**50**	40	10	0
**100**	75	20	5
**200**	150	40	10
**500**	375	100	25
**1000**	750	200	50

Supplementary Table 8. The breakup of each simulated keyword frequency used in the experiment investigating the linear range of SEEKER by a mixture of 1-keyword, 2-keyword and 5-keyword oligos.

6. Identifying potentially interfering sequences causing misidentification in SEEKER

File Number	Number of sequence fragment with penalty ≤ 5	Needleman–Wunsch alignment (Query, Fragment)	File Number	Number of sequence fragment with penalty ≤ 5	Needleman–Wunsch alignment (Query, Fragment)
**0**	3	GAGAG-CGGTGTGCGGTATCTG<br>GTGCGAC-GTGCGCGGTATCTG<br><br>GAG-AGCGGTGTGCGGTATCTG<br>-AGCAGTGGTGTGCGGTAGATG<br><br>GAGAGCGGTGTGCGGTATCTG<br>GAGAGCGGTGTATAGTAGCTA	**20**	0	
**1**	0		**21**	1	GAGAGC-GGTGTGCGGTATCTG<br>GAGATCAGATGTG-GATATCTG
**2**	1	GA-GAGCGGTGTGCGGTATCTG<br>GACG-GCAGTGTGCGCTATGTG	**22**	4	-GAGAGCGGTGTGCGGTATCTG<br>GGAGATATGTGTGCGGTATCT-<br><br>GAGAGCGGTGTGCGGTATCTG<br>GAGATATGTGTGCGGTATCTG<br><br>GAGAGCGGTGTGCGGTATCTG-<br>-AGATATGTGTGCGGTATCTGT<br><br>GAG-AGC-GGTGTGCGGTATCTG<br>-AGCA-CTCGTGTGCGGTATCTG
**3**	0		**23**	0	
**4**	3	-GAG-AGCGGTGTGCGGTATCTG<br>GGAGTA-TGGTGTGCGGTATCT-<br><br>GAG-AGCGGTGTGCGGTATCTG<br>GAGTA-TGGTGTGCGGTATCTG<br><br>GAG-AGCGGTGTGCGGTATCT-G<br>-AGTA-TGGTGTGCGGTATCTGG	**24**	0	
**5**	0		**25**	1	G-AGAGCGGTGTGCGGTATCTG<br>GTATAG-TGTGCGCGGTATCTG
**6**	0		**26**	1	GAGAGCGGTGTGCGGTA-TCTG<br>GCGCGCGGTGTGCGG-AGTATG
**7**	0		**27**	0	
**8**	2	GAGAG-CG-GTGTGCGGTATCTG<br>-AGCGTCGCGTGTGCGGTATCT-<br><br>GAG-AGCGGTGTGCGGTATCTG<br>GCGTCGC-GTGTGCGGTATCTG	**28**	0	
**9**	0		**29**	2	GAGAG-CG-GTGTGCGGTATCTG<br>-AGCGTCGCGTGTGCGGTATCT-<br><br>GAG-AGCGGTGTGCGGTATCTG<br>GCGTCGC-GTGTGCGGTATCTG
**10**	0		**30**	3	GAGAGC-GGTGTGCGGTATCTG<br>GCTA-CTGGTGCGCGGTATCTG<br><br>GAGA-GCGGTGTGCGGTATCTG<br>GTGATG-TGTGCGCGGTATCTG<br><br>GAGA-GCGGTGTGCGGTATCTG<br>GTGATG-TGTGCGCGGTATCTG
**11**	1	GAGAGCGGTGTGCGGTATC-TG<br>GAGAGTGATAT-CGGTATCATG	**31**	1	GAGAGC-GGTGTGCGGTATCTG<br>G-TAGCTAGTATGCGGTATCTG
**12**	1	GAGAGCGGTG-TGCGGTATCTG<br>GTCA-CGCTGATGCGGTATCTG	**32**	2	G-AGAGCGGTGTGCGGTATCT-G<br>GTAGCGCGGTGTGCGG-A-CTAG

					GAGAGCG-GT-GTGCGGTATCTG<br>-TGAGCGTGTAGT-CGGTATCTG
**13**	1	G-AGAGCGGTGTGCGGTATCTG<br>GTCGAG-TGTGCGCGGTATCTG	**33**	0	
**14**	0		**34**	0	
**15**	3	GAGAGCGGTGTGCGGTATCTG<br>GCTCGCAGTGTGCGGTATCTG<br><br>G-AGAGCGGTGTGCGGTATCTG<br>GTATA-CGGTGCGCGGTATCTG<br><br>GAGAGCGGTGTGCGGTATCT-G<br>TATA-CGGTGCGCGGTATCTGG	**35**	0	
**16**	0		**36**	8	G-AGAGCGGTGTGCGGTATCTG<br>GTATAGCGGCGTGCGG-ATCTA<br><br>GAG-A-GCGGTGTGCGGTATCTG<br>-AGTACGCGGTGCGCGGTATCT-<br><br>G-AGAGCGGTGTGCGGTATCTG<br>GTA-CGCGGTGCGCGGTATCTG<br><br>GAGAGCGGTGTGCGGTATCT-G<br>TA-CGCGGTGCGCGGTATCTGG<br><br>GAGAGCGGTGTGCGGTATCTG<br>TATAGCGGCGTGCGGTAGATG<br><br>GAG-A-GCGGTGTGCGGTATCTG<br>-AGTACGCGGTGCGCGGTATCT-<br><br>G-AGAGCGGTGTGCGGTATCTG<br>GTA-CGCGGTGCGCGGTATCTG<br><br>GAGAGCGGTGTGCGGTATCT-G<br>TA-CGCGGTGCGCGGTATCTGG
**17**	0		**37**	2	GAGAGCGGTGTGCGGTATCTG<br>GACTGTAGTGCGCGGTATCTG<br><br>GAG-AGCGGTGTGCGGTATCTG<br>GCGCTGC-GTGCGCGGTATCTG
**18**	0		**38**	0	
**19**	1	GAGAGCG-GTGTGCGGTATCTG<br>GCGA-CGTGTATGCGGTATCTG	**39**	2	GAGA-GCGGTGTGCGGTATCTG<br>GCGATAC-GTGCGCGGTATCTG<br><br>GAGAGC-GGTGTGCGGTATCTG<br>TCGA-CTGGTATGCGGTATCTG

Supplementary Table 9. List of potential interfering sequences that had a sum of gap penalty and mismatch penalty smaller than 5 after Needleman–Wunsch alignment between the query sequence corresponding to "abase_" and every 21-nt sequence fragment of the data strand in each file. "_" refers to a blank space. Sequences uniquely found in Files #35 and #36 were considered the source of misidentification and are marked in red. The list of comparison between the query "databa" and sequence fragments of the data strand is not presented because no sequences in Files #35 and #36 were found to be highly similar to the query sequence.

File Number	Number of sequence fragment with penalty ≤ 5	Needleman–Wunsch alignment (Query, Fragment)	File Number	Number of sequence fragment with penalty ≤ 5	Needleman–Wunsch alignment (Query, Fragment)
**0**	0		**20**	0	
**1**	0		**21**	0	
**2**	1	GACACGTGATCACG-GA-TCAGT<br>GACACGTGA-CATGTGACT-AGT	**22**	4	-GACACGTGATCACGG-ATCAGT<br>TGACACGTGATCACGGTA-CA-T<br><br>GACACGTGATCACGG-ATCAGT-<br>GACACGTGATCACGGTA-CA-TA<br><br>GACAC-GTGATCACGGA-TCAGT<br>GATACAGT-ATCACGGACTCAG-<br><br>ACTGATC-CGTGATCACGTGT-C<br>AGTGA-CACGTGATCACG-GTAC
**3**	0		**23**	0	
**4**	0		**24**	0	
**5**	8	--GACACGTGATCACGGATCAGT<br>TGGACACGTGATCACGGA-GA-T<br><br>-GACACGTGATCACGGATCAGT-<br>GGACACGTGATCACGGA-GA-TA<br><br>GACACGTGATCAC-G-GATCAGT<br>GACACGTGATCACGGAGAT-A-T<br><br>GACACGTGATCAC-G-GATCAGT<br>-ACACGTGATCACGGAGAT-ATT<br><br>--GACACGTGATCACGGATCAGT<br>TGGACACGTGATCACGGA-GA-T<br><br>-GACACGTGATCACGGATCAGT-<br>GGACACGTGATCACGGA-GA-TA<br><br>GACACGTGATCAC-G-GATCAGT<br>GACACGTGATCACGGAGAT-A-T<br><br>GACACGTGATCAC-G-GATCAGT<br>-ACACGTGATCACGGAGAT-ATT	**25**	1	ACTGA-TCCGTGA-TCACGTGTC<br>AC-GACTGCG-GAGTCACGTGTC
**6**	5	GACACGTGATCACG-GATCAGT<br>GACATGTG-TGACGAGATCAGA<br><br>--GACACGTGATCACGGATCAGT<br>CAGACACGTGATCACGGA-GA-T<br><br>-GACACGTGATCACGGATCAGT-<br>AGACACGTGATCACGGA-GA-TA<br><br>GACACGTGATCAC-G-GATCAGT<br>GACACGTGATCACGGAGAT-A-T<br><br>GACACGTGATCAC-G-GATCAGT<br>-ACACGTGATCACGGAGAT-ATT	**26**	5	--GACACGTGATCACGGATCAGT<br>CGGACACGTGATCACGG—CAGC<br><br>-GACACGTGATCACGGATCAG-T<br>GGACACGTGATCACGG—CAGCG<br><br>GACACGTGATCACGG-ATCAGT<br>GACACGTGATCACGGCAGC-GT<br><br>GACACGTGATCACGG-ATCAGT-<br>-ACACGTGATCACGGCAGC-GTG<br><br>GACACGTGA-TCACGGATCAGT<br>GACACGTGACTGACGTA-CAGT
**7**	0		**27**	7	--GACACGTGATCACGGATCAGT<br>TGGACACGTGATCACGGA-GA-T<br><br>-GACACGTGATCACGGATCAGT-<br>GGACACGTGATCACGGA-GA-TA<br><br>GACACGTGATCAC-G-GATCAGT<br>GACACGTGATCACGGAGAT-A-T

					GACACGTGATCACGG-ATCAGT<br>GACACGTGATCGCGGTGTC-GC<br><br>GACACGTGATCACGGATC-AG-T<br>GACACGTGATCGCGG—CGAGAT<br><br>ACT-GATCCGTGATCACGTGTC<br>ACTAGATCTATG-TCTCGTGTC<br><br>ACTGATC-CGTGATCAC-GTGTC<br>AC-GA-CACGTGATCGCGGTGTC
**8**	0		**28**	1	GACACGTGATCAC-GGATCAG-T<br>GACACGTTA-CACTGGA-CAGAT
**9**	5	--GACACGTGATCACGGATCAGT<br>TGGACACGTGATCACGG-TCAG-<br><br>-GACACGTGATCACGGATCAGT<br>GGACACGTGATCACGG-TCAGA<br><br>GACACGTGATCACGGATCAG-T<br>GACACGTGATCACGG-TCAGAC<br><br>GACACGTGATCACGGATCAG--T<br>-ACACGTGATCACGG-TCAGACG<br><br>ACT-GATC-CGTGATCACGTGTC<br>GCTGGA-CACGTGATCACG-GTC	**29**	0	
**10**	0		**30**	31	-GACACGTGATCACGG-ATCAGT<br>AGACACGTGATCACGGTATCA—<br><br>GACACGTGATCACGG-ATCAGT<br>GACACGTGATCACGGTATCA-T<br><br>GACACGTGATCACGG-ATCA-GT<br>-ACACGTGATCACGGTATCATG-<br><br>GACACGTGATCACGG-ATCA-GT<br>--CACGTGATCACGGTATCATGT<br><br>-GACACGTGATCACGG-ATCAGT<br>AGACACGTGATCACGGTATCA—<br><br>GACACGTGATCACGG-ATCAGT<br>GACACGTGATCACGGTATCA-T<br><br>GACACGTGATCACGG-ATCA-GT<br>-ACACGTGATCACGGTATCATG-<br><br>GACACGTGATCACGG-ATCA-GT<br>--CACGTGATCACGGTATCATGT<br><br>GACACGTGATCACG-GATCAGT<br>GACATGTG-TGACGAGATCAGT<br><br>-GACACGTGATCACGG-ATCAGT<br>GGACACGTGATCACGGTATCA—<br><br>GACACGTGATCACGG-ATCAGT<br>GACACGTGATCACGGTATCA-T<br><br>GACACGTGATCACGG-ATCA-GT<br>-ACACGTGATCACGGTATCATG-<br><br>GACACGTGATCACGG-ATCA-GT<br>--CACGTGATCACGGTATCATGT<br><br>-GACACGTGATCACGG-ATCAGT<br>CGACACGTGATCACGGTATCA—<br><br>GACACGTGATCACGG-ATCAGT<br>GACACGTGATCACGGTATCA-T

					GACACGTGATCACGG-ATCA-GT<br>-ACACGTGATCACGGTATCATG-<br><br>GACACGTGATCACGG-ATCA-GT<br>--CACGTGATCACGGTATCATGT<br><br>-GACACGTGATCACGG-ATCAGT<br>TGACACGTGATCACGGTATCA—<br><br>GACACGTGATCACGG-ATCAGT<br>GACACGTGATCACGGTATCA-T<br><br>GACACGTGATCACGG-ATCA-GT<br>-ACACGTGATCACGGTATCATG-<br><br>GACACGTGATCACGG-ATCA-GT<br>--CACGTGATCACGGTATCATGT<br><br>--GACACGTGATCACGGATCAGT<br>AGGACACGTGATCACGG-TCAG-<br><br>-GACACGTGATCACGGATCAGT<br>GGACACGTGATCACGG-TCAGA<br><br>GACACGTGATCACGGATCAG-T<br>GACACGTGATCACGG-TCAGAC<br><br>GACACGTGATCACGGATCAG--T<br>-ACACGTGATCACGG-TCAGACG<br><br>-GACACGTGATCACGG-ATCAGT<br>TGACACGTGATCACGGTATCA—<br><br>GACACGTGATCACGG-ATCAGT<br>GACACGTGATCACGGTATCA-T<br><br>GACACGTGATCACGG-ATCA-GT<br>-ACACGTGATCACGGTATCATG-<br><br>GACACGTGATCACGG-ATCA-GT<br>--CACGTGATCACGGTATCATGT<br><br>ACTGATC-CGTGATCAC-GTGTC<br>A-TGA-CACGTGATCACGGTATC<br><br>ACTGATC-CGTGATCAC-GTGTC<br>A-TGA-CACGTGATCACGGTATC
**11**	0		**31**	1	GACACGTGATCAC-GGATCAG-T<br>GACACGTTA-CACTGGA-CAGAT
**12**	1	GACACGTGATCAC-GGATCAG-T<br>GACACGTTA-CACTGGA-CAGAT	**32**	2	GA-CACGTGATCACG-GATCAGT<br>GAGCA-GTG-TGACGAGATCAGT<br><br>ACTGATC-CGTGA-TCACGTGTC<br>A-TG-TCGCGCGAGTCACGTGTC
**13**	7	GACACGTGATCACG-GATCAGT<br>TACGCGTG-TGACGAGATCAGT<br><br>-GACACGTGATCACGGATCAGT<br>CGACACGTGATCACGGA-GACT<br><br>GACACGTGATCACGGA-TCAGT<br>GACACGTGATCACGGAGACTG-<br><br>GACACGTGATCACGGA-TCAGT<br>-ACACGTGATCACGGAGACTGG<br><br>-GACACGTGATCACGGATCAGT<br>AGACACGTGATCACGGA-GACT	**33**	0	

		GACACGTGATCACGGA-TCAGT<br>GACACGTGATCACGGAGACTG-<br><br>GACACGTGATCACGGA-TCAGT<br>-ACACGTGATCACGGAGACTGG			
**14**	1	ACTGATCCGTGATCACGT-GTC<br>AATGGTGCGTGA-CACGTAGTC	**34**	1	GACACGTGATCACG-GATCAGT<br>GACGCGTGA-CGTGTGATCAGT
**15**	1	GACACGTGATC-ACGGATCAGT<br>GACACGTGAGCTAC-GCTCTGT	**35**	10	GACACGTGATC-ACG-GATCAGT<br>GACA-GTG-TCGTCGTGATCAGT<br><br>--GACACGTGATCACGGATCAGT<br>GTGACACGTGATCACGG-TCAG-<br><br>-GACACGTGATCACGGATCAGT<br>TGACACGTGATCACGG-TCAGA<br><br>GACACGTGATCACGGATCAG-T<br>GACACGTGATCACGG-TCAGAC<br><br>GACACGTGATCACGGATCAG--T<br>-ACACGTGATCACGG-TCAGACG<br><br>-GACACGTGATCACGG-ATCAGT<br>GGACACGTGATCACGGTA-CA-T<br><br>GACACGTGATCACGG-ATCAGT-<br>GACACGTGATCACGGTA-CA-TA<br><br>ACTGATC--CGTGATCACGTGTC<br>AGTG-TCGTCGTGATCA-GTGTC<br><br>AC-TGATCCGTGATCACGTGTC<br>TCGTGATCAGTG-TCTCGTGTC<br><br>AC-TGATC-CGTGATCACGTGTC<br>GCGTGA-CACGTGATCACG-GTC
**16**	0		**36**	5	GACACGTG-ATCACGGATCAG-T<br>GACGCGTGCA-CACGGAT-AGAT<br><br>--GACACGTGATCACGGATCAGT<br>TGGACACGTGATCACGGA-CA-T<br><br>-GACACGTGATCACGGATCAGT-<br>GGACACGTGATCACGGA-CA-TC<br><br>GACACGTGATCACGG--ATCAGT<br>GACACGTGATCACGGACATC-G-<br><br>GACACGTGATCACGG--ATCAGT<br>-ACACGTGATCACGGACATC-GG
**17**	2	GACACGTGATCACGG-ATCAGT<br>GATATGTGATCACGGCAGC-GT<br><br>GACACGTGA-TCACG-GATCAGT<br>GACACGTGACACACGCGA-C-GT	**37**	5	GACACGTGATC-ACG-GATCAGT<br>GACACGTGATCGACGTG-TC-GC<br><br>--GACACGTGATCACGGATCAGT<br>TGGACACGTGATCACGGA-GA-T<br><br>-GACACGTGATCACGGATCAGT-<br>GGACACGTGATCACGGA-GA-TA<br><br>GACACGTGATCAC-G-GATCAGT<br>GACACGTGATCACGGAGAT-A-T<br><br>ACTGATC-CGTGATC-ACGTGTC<br>AC-GA-CACGTGATCGACGTGTC
**18**	6	--GACACGTGATCACGGATCAGT<br>ATGACACGTGATCACGGA-GA-T<br><br>-GACACGTGATCACGGATCAGT-<br>TGACACGTGATCACGGA-GA-TA<br><br>GACACGTGATCAC-G-GATCAGT	**38**	0	

		GACACGTGATCACGGAGAT-A-T<br><br>GACACGTGATC-ACG-GATCAGT<br>GACA-GTG-TCGTCGTGATCAGT<br><br>ACTGATC--CGTGATCACGTGTC<br>AGTG-TCGTCGTGATCA-GTGTC<br><br>AC-TGATCCGTGATCACGTGTC<br>TCGTGATCAGTG-TCTCGTGTC			
**19**	0		**39**	0	

Supplementary Table 10. List of potential interfering sequences that had a sum of the gap penalty and mismatch penalty smaller than 5 after Needleman–Wunsch alignment between the query sequence corresponding to “comput” and every 21-nt sequence fragment of the data strand in each file. Sequences uniquely found in Files #35 and #36 were considered the source of misidentification and are marked in red.

File Number	Number of sequence fragment with penalty ≤ 5	Needleman–Wunsch alignment (Query, Fragment)	File Number	Number of sequence fragment with penalty ≤ 5	Needleman–Wunsch alignment (Query, Fragment)
**0**	0		**20**	3	-GTGTCGCGTGTCTGTGAGACA<br>AGTATCGCGTATCTG-GACACA<br><br>GTGTCGCGTGTCTGTGAGACA-<br>GTATCGCGTATCTG-GACACAG<br><br>GTGTCGCGTGTCTGTG-AGACA<br>GTGTCGCGAG-CTGTGTCGATA
**1**	3	-GTGTCGCGTGTCTGTGAGACA<br>AGTGTCTCACGTCTGTG-GACA<br><br>GTGTCGCGTGTCTGTGAGACA-<br>GTGTCTCACGTCTGTG-GACAC<br><br>GTGTCGCGTGTCTGT-GAGACA<br>GCGACGCGTGTCTGTGGTGAC-	**21**	0	
**2**	1	GTGTCGCGTGTCTGTG-AG-ACA<br>GTGT-GCG-GTCTGTGTAGTATA	**22**	2	-GTGTCGCGTGTCTGTGAGACA<br>GGTGTCGCGTGTCTG-GTGTCA<br><br>GTGTCGCGTGTCTGTGAGACA-<br>GTGTCGCGTGTCTG-GTGTCAT
**3**	0		**23**	0	
**4**	3	GTGTCGCGTGTC-TGTGAGACA<br>GAGACGCGTGTCATGTGAGA-T<br><br>GTGTCGCGTGTC-TGTGAGACA<br>-AGACGCGTGTCATGTGAGATA<br><br>GTGT-CGCGTG-TCTGTGAGACA<br>GTGTGCG-GTGCTCAGT-AGACA	**24**	3	GTGTC-G-CGTGTCTGTGAGACA<br>G-GTCAGACGTG-CAGTGAGACA<br><br>G-TGTCGCGTGTCTGTGAGACA<br>GACGACGCGTGTCTGTG-GACT<br><br>GTGTCGCGTGTCTGTGAGAC-A<br>ACGACGCGTGTCTGTG-GACTA
**5**	6	-GTGTCGCGT-GTCTGTGAGACA<br>CGTGTCGC-TCGTC-GTGAGATA<br><br>GTGTCGCGT-GTCTGTGAGACA-<br>GTGTCGC-TCGTC-GTGAGATAT<br><br>GTGTCGCGTGTC-TGTGAGACA<br>GAGACGCGTGTCATGTGAGA-T<br><br>GTGTCGCGTGTC-TGTGAGACA<br>-AGACGCGTGTCATGTGAGATA<br><br>GTGTCGCGTGT-CTGTGAGACA<br>GTGACGCGTGTGATGTG-TACA<br><br>TGTCTCA-CAGACACGCGACAC<br>TGTCACATTAGACACGAGACA-	**25**	0	
**6**	6	GTGTCGCGTGTCT-GTGAGACA<br>GTCTCGTGTCTCTAGT-AGACA<br><br>GTGTCGCGT-GTCTGTGAGACA<br>GTGTCGCGTAGTCGGTG-CTCA<br><br>GT-GTCGCGT-GTCTGTGAGACA<br>GTAGTCG-GTAGTGTGT-AGACA<br><br>GTGTCGCGTGT-CTGTGAGACA<br>GTGTCGAGTATACAGTGAG-CA<br><br>-GTGTCGCGTGTCTGTGAGACA<br>TGCGTCTCGTGTCTCT-AGACA<br><br>GTGTCGCGTGTCTGTGAGACA-	**26**	5	GT-GTCGCGTGTCTGTGAGACA<br>GTCGTCACG-ATATGTGAGACA<br><br>-GTGTCGCGTGTCTGTGAGACA<br>TGTGTCGCGTGTCTG-GTGTCA<br><br>GTGTCGCGTGTCTGTGAGACA-<br>GTGTCGCGTGTCTG-GTGTCAT<br><br>GTGTCG-CGTGTCTGT-GAGACA<br>G-GACGTCG-GTCTGTGGAGACA<br><br>GTGTCGCGTGTCTGTGAG-ACA<br>GTGTCGCATGTC-ATCAGTACA

		GCGTCTCGTGTCTCT-AGACAC			
**7**	0		**27**	1	GTGTCGCGTGTCTGT-GAGACA<br>G-GACGCGTGTCTGTGGTGACT
**8**	4	GTGTCGCGTGTC-TGTGAGACA<br>GAGACGCGTGTCATGTGAGA-T<br><br>GTGTCGCGTGTC-TGTGAGACA<br>-AGACGCGTGTCATGTGAGATA<br><br>-GTGTCGCGTGTCTGTGAGACA<br>GGTGTCGCGAGACTG-GCGACA<br><br>GTGTCGCGTGTCTGTGAGACA-<br>GTGTCGCGAGACTG-GCGACAC	**28**	0	
**9**	0		**29**	0	
**10**	1	GTGTCGCGTG-TCTGTGAGACA<br>GCGTCGCGAGATATGTGAG-CA	**30**	0	
**11**	0		**31**	4	GTGTCGCGTGT-CTGTGAGACA<br>GTGACGCGTGTGCTGCGAGAC-<br><br>GTGTCGCGTGT-CTGTGAGACA<br>-TGACGCGTGTGCTGCGAGACG<br><br>GTGTCGCGTGT-CTGTGAGACA<br>G-GACGCGTGTGCTGCGAGACG<br><br>GTGTCGCGTGT-CTGTGAGACA<br>G-GACGCGTGTGCTGCGAGACG
**12**	0		**32**	2	GT-GTCGCGTGTCTGTGA-GACA<br>-TCGTCGCATGTCTGT-ACGACA<br><br>GT-GTCGCGTGTCTGTGA-GACA<br>-TCGTCGCATGTCTGT-ACGACA
**13**	4	GTGTCGCGTGTCTGTGAGACA<br>CTGTCGCATGTCTACGTGACA<br><br>GTGTCGCGTG-TCTGTGAGACA<br>GTGTCGCGTGCTATGT-CGACG<br><br>GTGTCGCGTGTCT-GTGAGACA<br>GTGTCGTGTGACTAGT-AGACA<br><br>TGTCTCACAGACACGCGAC-AC<br>TGACTGACAG-CACACGACGAC	**33**	0	
**14**	0		**34**	4	GTGTCGCGTGTCTGTGAGAC-A<br>GTGTCGCGTATC-ATGACACTA<br><br>GTGTCGCGTGTC-TGTGAGACA<br>GTGTCGCATGTCATCAGAG-CA<br><br>GTGTCGCGTGTCT-GTGAGAC-A<br>GTGTCGC-TGTCTAGTG-TACTA<br><br>GTGTCGCGTGTC-TGTGAGACA<br>GTGACGCGTGACGTGTGA-TCA
**15**	6	GT-GTCGCGTGTCT-GTGAGACA<br>GTAGTCGCGCGTCTCGTGAG—A<br><br>GTGTCGCGTGTCT-GTGAGACA<br>-AGTCGCGCGTCTCGTGAGAGA<br><br>GTGTCGCGTGTCT-GTGAGACA<br>GTGTAGTGTATCTGGT-AGACA<br><br>-GTGTCGCGT-GTCTGTGAGACA<br>AGTGTCGC-TCGTC-GTGAGATA<br><br>GTGTCGCGT-GTCTGTGAGACA-<br>GTGTCGC-TCGTC-GTGAGATAT	**35**	7	-GTGTCGCGTGT-CTGTGAGACA<br>TGTGTCGCGTGTGCGGTGAG—A<br><br>GTGTCGCGTGT-CTGTGAGACA<br>GTGTCGCGTGTGCGGTGAGA-T<br><br>GTGTCGCGTGT-CTGTGAGACA<br>-TGTCGCGTGTGCGGTGAGATG<br><br>-GTGTCGCGT-GTCTGTGAGACA<br>CGTGTCGC-TCGTC-GTGAGATA<br><br>GTGTCGCGT-GTCTGTGAGACA-<br>GTGTCGC-TCGTC-GTGAGATAT

		T-GTCTCACAGACACGCGACAC<br>TGGTCT-GCAGTCGCGCGACAC			-GTGTCGCGTGTCTGTGAGACA<br>TGTGTCGCGAGACTG-GAGACA<br><br>GTGTCGCGTGTCTGTGAGACA-<br>GTGTCGCGAGACTG-GAGACAT
**16**	0		**36**	16	-GTGTCGCGTGTCTGTGAGACA<br>GGTGTCGC-TCTCTGCGAGATA<br><br>GTGTCGCGTGTCTGTGAGACA-<br>GTGTCGC-TCTCTGCGAGATAT<br><br>-GTGTCGCGTGTCTGTGAGACA<br>CGCGTCGCATGTCTGTGAGA-T<br><br>GTGTCGCGTGTCTGTGAGA-CA<br>GCGTCGCATGTCTGTGAGATC-<br><br>GTGTCGCGTGTCTGTGAGA-CA<br>-CGTCGCATGTCTGTGAGATCT<br><br>GTGTCGCGTGTCTGTGAGA-C-A<br>--GTCGCATGTCTGTGAGATCTA<br><br>GTGTCGCGTG-TCTGTGAGACA<br>GCGTCGCGTGCTCAGT-AGACA<br><br>GTGTCGCGTG-TCTGTGAGACA<br>GTCACGCGTGCTCAGT-AGACA<br><br>G-TGTCGCGTGTCTG-TGAGACA<br>GATGTCTCGTGTC-GCTGAG-CA<br><br>-GTGTCGCGTGTCTGTGAGACA<br>AGTGTCGCGTGTCTGT-ATAGA<br><br>GTGTCGCGTGTCTGT-GAGACA<br>GTGTCGCGTGTCTGTATAGAC-<br><br>GTGTCGCGTGTCTGT-GAGACA<br>-TGTCGCGTGTCTGTATAGACG<br><br>GT-GTCGCGTGTCTGTGAGACA<br>ATGGACGCGTGTCTGTGAGA-T<br><br>-GTGTCGCGTGTCTGTGAGA-CA<br>TG-GACGCGTGTCTGTGAGATC-<br><br>GTGTCGCGTGTCTGTGAGA-CA<br>G-GACGCGTGTCTGTGAGATCT<br><br>GTGTCGCGTGTCTGTGAGA-C-A<br>--GACGCGTGTCTGTGAGATCTA
**17**	5	-GTGTCGCGTGTCTGTGAGACA<br>AGTATCGCGTATCTG-GAGAGA<br><br>GTGTCGCGTGTCT-GTGAGACA<br>GTATCGCGTATCTGGAGAGAC-<br><br>GTGTCGCGTGTC-TGTGAGACA<br>GACTCGCGTGTCATGTGAGA-T<br><br>GTGTCGCGTGTC-TGTGAGACA<br>-ACTCGCGTGTCATGTGAGATA<br><br>TGTCTCACA-GACACGCGACAC<br>TGTGTGACACGTCACGCG-CAC	**37**	4	GTGTCG-CGTGTCTGT-GAGACA<br>GTGTCGTCGTGTCAGTCGA-AC-<br><br>GTGTCGCGTG-TCTGTGAGACA<br>GTGTCGCGAGATATGTCAGAC-<br><br>GTGTCGCGTG-TCTGTGAGACA<br>-TGTCGCGAGATATGTCAGACA<br><br>GTGTCGCGTGTCTGTG-A-GACA<br>GTGTCGCG-CTCTGTGTATGAC-
**18**	4	-GTGTCGCGTGTCTGTGAGACA<br>GGTGTCGCGTGTCTG-GTGTCA<br><br>GTGTCGCGTGTCTGTGAGACA-<br>GTGTCGCGTGTCTG-GTGTCAT	**38**	0	

		GTGTCGCGTGTCTGT-GAGACA<br>G-GTCGCGCGACTGTAGAGATA<br><br>TGTCTCACAG-ACACGCGACAC<br>TGTCTC-TAGTACACGCGTCGC			
**19**	2	GT-GTCGCGTGTCTGTGA-GACA<br>-TCGTCGCATGTCTGT-ACGACA<br><br>GTGTCGCGTGTCTGTG-AGACA<br>GTGTCGCGTGTCTG-GCTCACA	**39**	3	-GTGTCGCGTGTCTGTGAGAC-A<br>TGTGTCGCGTGTCTG-GA-TCGA<br><br>GTGTCGCGTGTCTGTGA--GACA<br>GTGTCGCGTGTCTG-GATCGAC-<br><br>GTGTCGCGTGTCTGTGA--GACA<br>-TGTCGCGTGTCTG-GATCGACG

Supplementary Table 11. List of potential interfering sequences that had a sum of the gap penalty and mismatch penalty smaller than 5 after Needleman–Wunsch alignment between the query sequence corresponding to "_compu" and every 21-nt sequence fragment of the data strand in each file. "_" refers to a blank space. Sequences uniquely found in Files #35 and #36 were considered the source of misidentification and are marked in red.

References

1 Goldman, N. *et al.* Towards practical, high-capacity, low-maintenance information storage in synthesized DNA. *Nature 2013 494:7435* **494**, 77-80 (2013). https://doi.org:10.1038/nature11875

2 Kosuri, S. & Church, G. M. Large-scale de novo DNA synthesis: technologies and applications. *Nat Methods* **11**, 499-507 (2014). https://doi.org:10.1038/nmeth.2918

3 Erlich, Y. & Zielinski, D. DNA Fountain enables a robust and efficient storage architecture. *Science* **355**, 950-954 (2017). https://doi.org:10.1126/SCIENCE.AAJ2038/SUPPL_FILE/ERLICH.SM.PDF

4 Untergasser, A. *et al.* Primer3—new capabilities and interfaces. *Nucleic Acids Research* **40**, e115 (2012). https://doi.org:10.1093/NAR/GKS596

5 Koressaar, T. & Remm, M. Enhancements and modifications of primer design program Primer3. *Bioinformatics (Oxford, England)* **23**, 1289-1291 (2007). https://doi.org:10.1093/BIOINFORMATICS/BTM091

6 Organick, L. *et al.* Random access in large-scale DNA data storage. *Nature Biotechnology 2018 36:3* **36**, 242-248 (2018). https://doi.org:10.1038/nbt.4079

7 Quail, M. A. *et al.* A tale of three next generation sequencing platforms: comparison of Ion Torrent, Pacific Biosciences and Illumina MiSeq sequencers. *BMC genomics* **13** (2012). https://doi.org:10.1186/1471-2164-13-341
