# Supplementary material for: CRISPR-powered quantitative keyword search engine in DNA data storage
Source: Nat Commun. 2024 Mar 15;15:2376. doi: 10.1038/s41467-024-46767-x (PMC10943086; doi:10.1038/s41467-024-46767-x)
Supplement: Supplementary file 2 — Reporting Summary [file 41467_2024_46767_MOESM2_ESM.pdf]

nature portfolio

Corresponding author(s): Changchun Liu

Last updated by author(s): 03/03/2024

Reporting Summary

Nature Portfolio wishes to improve the reproducibility of the work that we publish. This form provides structure for consistency and transparency in reporting. For further information on Nature Portfolio policies, see our Editorial Policies and the Editorial Policy Checklist.

Statistics

For all statistical analyses, confirm that the following items are present in the figure legend, table legend, main text, or Methods section.

n/a	Confirmed	
☐	☒	The exact sample size (*n*) for each experimental group/condition, given as a discrete number and unit of measurement
☐	☒	A statement on whether measurements were taken from distinct samples or whether the same sample was measured repeatedly
☒	☐	The statistical test(s) used AND whether they are one- or two-sided<br>*Only common tests should be described solely by name; describe more complex techniques in the Methods section.*
☒	☐	A description of all covariates tested
☐	☒	A description of any assumptions or corrections, such as tests of normality and adjustment for multiple comparisons
☐	☒	A full description of the statistical parameters including central tendency (e.g. means) or other basic estimates (e.g. regression coefficient) AND variation (e.g. standard deviation) or associated estimates of uncertainty (e.g. confidence intervals)
☒	☐	For null hypothesis testing, the test statistic (e.g. *F*, *t*, *r*) with confidence intervals, effect sizes, degrees of freedom and *P* value noted<br>*Give P values as exact values whenever suitable.*
☒	☐	For Bayesian analysis, information on the choice of priors and Markov chain Monte Carlo settings
☒	☐	For hierarchical and complex designs, identification of the appropriate level for tests and full reporting of outcomes
☒	☐	Estimates of effect sizes (e.g. Cohen's *d*, Pearson's *r*), indicating how they were calculated

Our web collection on statistics for biologists contains articles on many of the points above.

Software and code

Policy information about availability of computer code

Data collection	Real-time fluorescence was measured by CFX96 Touch Real-Time PCR Detection System (Bio-Rad Laboratories). The fluorescence images of tubes and chips were obtained through the ChemiDoc MP Imaging System (Bio-Rad Laboratories) or a blue light LED illuminator. Sequencing data was generated through Illumina MiSeq System.
Data analysis	Matlab R2022b was used to analyze results and produce Supplementary Fig. 3a-b and Supplementary Fig. 4d. All other figures showing experimental or analytical results were produced with OriginPro 2022b. The source code is written in Python 3 and is publicly available on GitHub repository at https://github.com/Jiozhang/SEEKER-encoding-and-decoding. All packages and dependencies required to execute the code are listed in "requirements.txt". The primary packages required to implement the code include primer3 and reedsolo.

For manuscripts utilizing custom algorithms or software that are central to the research but not yet described in published literature, software must be made available to editors and reviewers. We strongly encourage code deposition in a community repository (e.g. GitHub). See the Nature Portfolio guidelines for submitting code & software for further information.

Data

Policy information about availability of data

All manuscripts must include a data availability statement. This statement should provide the following information, where applicable:

- Accession codes, unique identifiers, or web links for publicly available datasets
- A description of any restrictions on data availability
- For clinical datasets or third party data, please ensure that the statement adheres to our policy

The source data underlying Figs. 3–5, Supplementary Figs. 3-4, 6, and 15–24 in this study are provided in the Source Data file. Matlab R2022b was used to analyze results and produce Supplementary Fig. 3a-b and Supplementary Fig. 4d. All other figures showing experimental or analytical results were produced with OriginPro 2022b. The raw sequencing reads of the reference pool and data pools #1–4 in FASTQ format are publicly available in "Raw reads.zip" file at https://github.com/Jiozhang/SEEKER-encoding-and-decoding. Source data are provided with this paper.

Research involving human participants, their data, or biological material

Policy information about studies with human participants or human data. See also policy information about sex, gender (identity/presentation), and sexual orientation and race, ethnicity and racism.

Reporting on sex and gender	N/A
Reporting on race, ethnicity, or other socially relevant groupings	N/A
Population characteristics	N/A
Recruitment	N/A
Ethics oversight	N/A

Note that full information on the approval of the study protocol must also be provided in the manuscript.

Field-specific reporting

Please select the one below that is the best fit for your research. If you are not sure, read the appropriate sections before making your selection.

☒ Life sciences ☐ Behavioural & social sciences ☐ Ecological, evolutionary & environmental sciences

For a reference copy of the document with all sections, see nature.com/documents/nr-reporting-summary-flat.pdf

Life sciences study design

All studies must disclose on these points even when the disclosure is negative.

Sample size	No sample size calculation was performed. In multi-file SEEKER experiment, the synthetic oligo pool contains 40 files where each file contains 50 oligos on average, storing the information of ~40 KB text data from 40 abstracts of journal articles. This sample size is sufficient as it comprises ~8,000 irrelevant background words as interferences for searching, which we believe has never been demonstrated before and is large enough to test the stability of the developed system.
Data exclusions	No data was excluded from this study.
Replication	For SEEKER experiment, we performed 160 individual searches in total on 4 queries to demonstrate the feasibility and reliability of searching. For the analysis of minimum sequencing coverage for full recovery of data, we performed 3 repeats of 20 independent sub-sampling experiments on the sequencing reads. All attempts at replication were successful.
Randomization	N/A as samples were all in vitro samples.
Blinding	N/A as samples were all in vitro samples.

Reporting for specific materials, systems and methods

We require information from authors about some types of materials, experimental systems and methods used in many studies. Here, indicate whether each material, system or method listed is relevant to your study. If you are not sure if a list item applies to your research, read the appropriate section before selecting a response.

Materials & experimental systems

n/a	Involved in the study
[x]	[ ] Antibodies
[x]	[ ] Eukaryotic cell lines
[x]	[ ] Palaeontology and archaeology
[x]	[ ] Animals and other organisms
[x]	[ ] Clinical data
[x]	[ ] Dual use research of concern
[x]	[ ] Plants

Methods

n/a	Involved in the study
[x]	[ ] ChIP-seq
[x]	[ ] Flow cytometry
[x]	[ ] MRI-based neuroimaging
