# Supplementary material for: CRISPR-powered quantitative keyword search engine in DNA data storage
Source: Nat Commun. 2024 Mar 15;15:2376. doi: 10.1038/s41467-024-46767-x (PMC10943086; doi:10.1038/s41467-024-46767-x)
Supplement: Supplementary file 4 — Description of Additional Supplementary Files [file 41467_2024_46767_MOESM4_ESM.pdf]

Description of Additional Supplementary Files

Title: **Supplementary Data 1**

Description: Text content of File A and File B for single-file oligo pools

Title: **Supplementary Data 2**

Description: Text content of 40 randomly selected journal abstracts for multi-file oligo pool

Title: **Supplementary Data 3**

Description: PCR primer sequences for selectively amplifying multi-file oligo pool

Title: **Supplementary Data 4**

Description: Oligo sequences in single-file oligo pool

Title: **Supplementary Data 5**

Description: Reference oligo sequences in multi-file oligo pool

Title: **Supplementary Data 6**

Description: Data oligo sequences in multi-file oligo pool

Title: **Supplementary Data 7**

Description: crRNA dual query sequences

Title: **Supplementary Data 8**

Description: Files in non-text format used in Supplementary Fig. 4e and f
